# High-copy plasmid engineering enhances recombinant protein and antimicrobial peptide production in *Corynebacterium glutamicum*

**DOI:** 10.1186/s12934-026-03004-y

**Published:** 2026-04-13

**Authors:** Jens Christmann, Annalena Sommer, Peng Cao, Michael Kohlstedt, Oliver Goldbeck, Christian U. Riedel, Judith Becker, Christoph Wittmann

**Affiliations:** 1 Institute for Systems Biotechnology, Saarland University, Saarbrücken, Germany; 2 Institute of Microbiology and Biotechnology, University of Ulm, Ulm, Germany

**Keywords:** *Corynebacterium glutamicum*, Pediocin PA-1, Bioprocess, Food additive, Bacteriocin, Antimicrobial peptide, Plasmid copy number, Expression system, Peptide production, mCherry, *Listeria spp*

## Abstract

**Background:**

Antimicrobial peptides (AMPs) such as pediocin PA-1 are attractive for food biopreservation and infection control, but their broader use is limited by low recombinant yields and high production costs. *Corynebacterium glutamicum* has emerged as a robust GRAS chassis for heterologous peptide and protein production, yet commonly used shuttle vectors provide only moderate plasmid copy numbers and expression capacities. In particular, existing pediocin PA-1 processes in *C. glutamicum* rely on standard pBL1*-* or pCG1-family vectors that do not yet leverage replication-origin engineering.

**Results:**

We rationally redesigned the replication control region of the widely used pClik 5α (pCG1-family) backbone by introducing targeted mutations in the *repA* gene, an antisense RNA (*cgrI*) promoter, and putative partitioning genes *parAB*, and constructed a systematic panel of high-copy variants. Using a *P*_*tuf*_-driven mCherry reporter as a quantitative readout, we identified plasmids that supported several-fold higher fluorescence than the parental backbone while maintaining robust growth. Fluorescence-based gene-dosage estimation indicated a strong increase in apparent plasmid copy number. Independent qPCR-based plasmid copy number determination using two plasmid loci confirmed that the lead variant pClik 5α *repA*^*mut*^ reached approximately 28–30 copies per chromosome equivalent, compared to approximately 2–3 copies for the parental plasmid, corresponding to an approximately 10-fold increase. Genome-wide transcriptome analysis revealed a defined and adaptive transcriptional response to elevated plasmid copy number and expression burden, characterized by adjustments in membrane-associated transport, respiratory functions, and amino acid-related metabolism, without evidence of collapse of core biosynthetic functions. When the best-performing replicon was applied to episomal expression of a codon-optimized *pedACD*^*Cgl*^ operon, pediocin PA-1 titers increased by 2.5-fold compared to the best pXMJ19-based reference under identical, previously optimized process conditions, placing the system, under comparable cultivation formats, within the upper range of reported microbial pediocin production processes.

**Conclusions:**

This work demonstrates that rational engineering of pCG1-family replication modules in *C. glutamicum* can unlock markedly higher plasmid copy numbers and expression capacities while preserving physiological robustness. The resulting high-copy pClik 5α derivatives, exemplified by pClik 5α *repA*^*mut*^, provide a versatile high-copy expression platform with demonstrated utility for recombinant reporter protein and antimicrobial peptide production in *C. glutamicum* and offer a foundation for further integration with folding, secretion, and process engineering strategies to advance industrial AMP production.

**Supplementary Information:**

The online version contains supplementary material available at 10.1186/s12934-026-03004-y.

## Background

Recombinant proteins and antimicrobial peptides (AMPs) represent an increasingly important class of bioactive molecules with applications across biotechnology, medicine, and food safety. AMPs comprise more than 3,000 naturally occurring molecules identified across diverse taxa, where they serve as key components of innate immunity [1]. Their broad activities and robustness make them attractive candidates for combatting multidrug-resistant pathogens [2, 3] and for complementing the development of novel antibiotics [4]. In parallel, many recombinant peptides and proteins—including fluorescent reporters, industrial enzymes, and therapeutic scaffolds—depend on efficient microbial production platforms for cost-effective and scalable manufacturing [5].

Despite their potential, many AMPs remain difficult to synthesize or purify in quantities sufficient for biochemical and preclinical evaluation. This holds especially true for class IIa bacteriocins, a prominent subgroup characterized by strong activity against *Listeria* species [6]. Among these, pediocin PA-1 is the most extensively studied representative, valued for its high potency and favorable safety profile [7–9]. Yet, pediocin PA-1 is currently available only as a chemically synthesized peptide in limited quantities and at high cost [10], which restricts its use in systematic dose–response studies, formulation development, and larger-scale functional or preclinical evaluations [11]. Sequencing of the *pedABCD* operon in *Pediococcus acidilactici* enabled heterologous production in various microbial hosts [12–15], but yields have typically remained insufficient for broader application. In parallel, earlier studies in lactic acid bacteria (LAB), *Bacillus subtilis*, and *E. coli* established pediocin PA-1 as a tractable yet challenging target, demonstrating secretion strategies, leader processing, and structural determinants of activity [16–18]. 

*Corynebacterium glutamicum* has emerged as a robust chassis for recombinant peptide and protein production, including pediocin PA-1, via expression of a codon-optimized version of *pedACD* [19]. Long established as an industrial amino acid producer, *C. glutamicum* offers GRAS status, compatibility with defined media, and a favorable safety profile due to the absence of endotoxins and extracellular proteases [20–22]. Efficient episomal expression systems have enabled high-level production of diverse heterologous proteins, including fluorescent reporters such as mCherry and GFP [23, 24], establishing *C. glutamicum* as a promising host for expanding accessible recombinant peptide portfolios.

Key to maximizing productivity in *C. glutamicum* is the performance of its plasmid vectors. Common shuttle vectors combine origins of replication (ORI) from both *Escherichia coli* and *C. glutamicum*, along with antibiotic markers for dual-host cloning and selection [25]. The *C. glutamicum* replication modules are derived primarily from cryptic plasmids of the pBL1 [26] and pCG1 families [27], systematically classified [28] to replicate via a rolling-circle mechanism initiated by the essential replication protein RepA [28]. Plasmid copy number and stability are highly sensitive to the sequence composition of the replication locus. Specific RepA mutations [29], antisense-RNA–based regulation (e.g. *cgrI*) and the presence or absence of partitioning systems such as *parAB* [30] all modulate plasmid abundance and performance. Even single amino acid changes in RepA can substantially alter copy numbers and affect recombinant protein expression [29]. These insights highlight the replication machinery as a powerful engineering lever to enhance plasmid-encoded product yields.

Over the past decades, extensive work has established a rich genetic toolbox for *C. glutamicum*, including pBL1- and pCG1-family shuttle vectors, promoter libraries, tunable expression systems, and genome-integration platforms [30–37]. These tools have enabled a wide range of heterologous protein productions—from fluorescent reporters to industrial enzymes—and laid the foundation for recombinant pediocin PA-1 biosynthesis in this host [19, 38]. Despite these advances, plasmid replication engineering remains comparatively underexplored in *C. glutamicum*. Notably, no prior study has systematically engineered pCG1-family replication origins to define tunable copy-number ranges or evaluated their genome-wide physiological consequences. Addressing this gap, our study combines replication-origin engineering, high-throughput reporter screening, and genome-wide transcriptome analysis to develop next-generation high-copy plasmids and evaluate their impact on recombinant protein and pediocin PA-1 yields in *C. glutamicum*.

To enable systematic screening of replication variants, we employed mCherry as a quantitative reporter whose fluorescence reflects plasmid abundance and expression capacity, providing a scalable proxy for evaluating high-copy vectors that support robust protein and peptide production. This relationship holds under non-saturating expression conditions, as shown previously for *C. glutamicum* reporter systems [34]. Previous work on pediocin PA-1 production in *C. glutamicum* relied primarily on conventional vectors such as pXMJ19 [19], which leaves considerable room for improvement in terms of plasmid copy number, stability, and expression capacity. Motivated by this, we sought to optimize plasmid-driven production through systematic engineering of the replication control region of the widely used pClik 5α backbone [39, 40], which originates from early *C. expression* systems described in foundational work [41] and related patent developments [42]. A targeted library of ORI variants was constructed and evaluated using mCherry fluorescence as a quantitative proxy for expression strength, enabling the identification of stably maintained high-copy plasmids with enhanced performance. To understand cellular adaptation to the increased plasmid and expression burden, transcriptome analysis was applied. Finally, the best-performing plasmid variant, pClik 5α *repA*^*mut*^, was applied to the production of pediocin PA-1, yielding a 2.5-fold improvement over previously reported pXMJ19-based systems under identical conditions. Together, this work advances the mechanistic understanding of replication control in *C. glutamicum*, provides a valuable family of high-copy expression vectors, and supports the broader use of this industrially established microorganism for efficient recombinant protein and peptide production.

To our knowledge, no study has so far systematically modified the replication-control region of the widely used pCG1-family plasmid pClik 5α to expand its accessible copy-number range or linked these variants to global transcriptional responses and AMP production performance.

## Results

### Rational engineering of high-copy pClik 5α plasmids for *C. glutamicum*

To increase plasmid-based expression capacity in *C. glutamicum*, we rationally engineered the widely used shuttle vector pClik 5α by targeting key elements of its replication control region. Based on previous work on pCG1-family plasmids, we focused on the replication initiator gene *repA*, the adjacent partitioning genes *parAB*, and regulatory sequences implicated in antisense RNA–mediated copy number control. On this basis, we generated a panel of pClik 5α derivatives carrying (i) the RepA G429E copy number mutation (*repA*^mut^), (ii) a deletion of the putative partitioning module (Δ*parAB*), in which *parA* and *parB* were removed simultaneously to assess the combined effect of this region, (iii) a mutation in the promoter of the antisense RNA *cgrI* (*cgrI*^mut^), and (iv) all possible combinations thereof (Table 1). All plasmid variants were constructed from the pClik 5α backbone and verified by restriction analysis and Sanger sequencing of the modified regions. The resulting plasmid family maintained an identical selection marker and expression cassette architecture across constructs, such that functional differences could be attributed to the engineered replication control elements. A schematic overview of all engineered ORI modifications, including *repA*^*mut*^, *cgrI*^*mut*^, and *ΔparAB*, is provided in Fig. S1 (Additional File 1).

As host, we employed the prophage-free, genome-reduced strain *C. glutamicum* CR099, derived from ATCC 13,032 and lacking the cryptic prophages CGP1–3 and insertion sequences ISCg1–2 [43], providing a stable chassis for plasmid-based production (Table 1). To enable quantitative comparison of plasmid performance, we equipped each pClik 5α variant with a constitutively expressed *mCherry* reporter gene under control of the *P*_*tuf*_ promoter [44]. Because mCherry fluorescence correlates linearly with gene dosage under non-saturating expression conditions, it provides a reliable proxy for estimating relative plasmid abundance. In addition, mCherry has been shown to exhibit low background interference and high signal specificity in *C. glutamicum*, in contrast to alternative reporters such as eGFP, which can be affected by cellular autofluorescence [45]. The cassette was integrated at the same locus within the multiple cloning site of each plasmid, ensuring a standardized expression context. In addition to the pClik 5α-based variants, we constructed pXMJ19 Δ*P*_*tac*_ *P*_*tuf*_ *mCherry*, which lacks the inducible *P*_*tac*_ promoter and the associated *lacI* repressor. This modification enabled *mCherry* expression solely under the constitutive *P*_*tuf*_ promoter, providing a promoter-normalized control for benchmarking plasmid-driven expression independently of copy number effects (Table 1). Following assembly and sequence validation, pXMJ19 Δ*P*_*tac*_ *P*_*tuf*_ *mCherry* was transformed into CR099 alongside the pClik 5α derivatives. Finally, we constructed the reference strain CR099::*P*_*tuf*_ *mCherry*, in which a single *mCherry* copy is integrated into the chromosome, providing a benchmark for estimating relative plasmid copy numbers (Table 1). All engineered pClik 5α derivatives carrying *P*_*tuf*_ *mCherry* were successfully transformed into CR099, and transformants were obtained at robust frequencies, indicating that none of the modifications abolished plasmid replication or maintenance. Under standard preculture conditions, strains harboring the parental pClik 5α plasmid or its derivatives exhibited only moderate growth differences, with reduced growth rates and extended growth phases observed for high-copy variants.

**Fig. 1:**
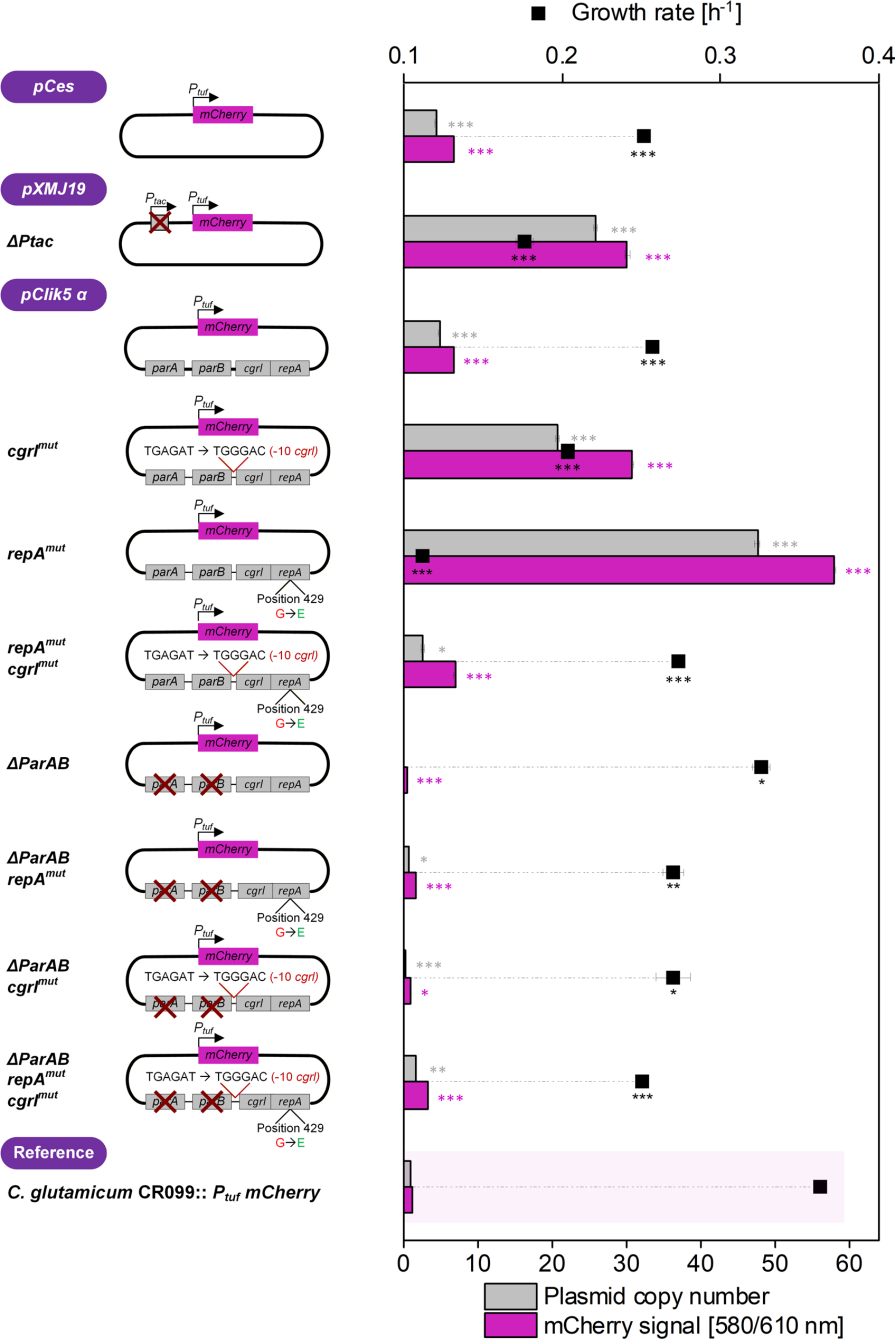
Screening of episomal expression vectors variants for copy number based on expression of the mCherry reporter. Episomal expression of Ptuf-driven mCherry in *C. glutamicum* CR099 was evaluated using the comparator plasmids pCes *P*_*tuf*_ *mCherry* and pXMJ19 Δ*P*_*tac*_ *P*_*tuf*_ *mCherr*y, together with a panel of engineered pClik 5α variants carrying targeted modifications in the replication region. Cultivations were performed under uniform conditions (1300 rpm, 30 °C) in a miniaturized microtiter microbioreactor with online monitoring of optical density (620 nm) and mCherry fluorescence (excitation/emission = 580/610 nm). The chromosomal reference strain CR099::P_*tuf*_ *mCherry*, containing a single genomic copy of *mCherry*, served to benchmark plasmid-derived expression and approximate relative copy number differences. Data represent biological duplicates (*n* = 2). Statistical significance was assessed using an unpaired two-sided t-test relative to the chromosomal reference strain CR099::*P*_*tuf*_ *mCherry*. Significance levels are indicated as follows: * *p* < 0.05, ** *p* < 0.01, *** *p* < 0.001. Statistical analysis was performed for mCherry fluorescence, plasmid copy number, and growth rate

In summary, we established a coherent library of pClik 5α-based expression plasmids with systematically varied replication control regions and a standardized *P*_*tuf*_ *mCherry* reporter module in the common host background CR099 (Table 1). This strain–plasmid panel formed the basis for fluorescence-based screening of copy-number variants, in which mCherry production was employed as a proxy for plasmid abundance and expression capacity suitable for systematic copy-number screening (Fig. 1).

### High-throughput mCherry screening identifies high-expression pClik 5α variants

To functionally compare the engineered plasmid family, we quantified mCherry production as a proxy for plasmid abundance and expression efficiency. *C. glutamicum* CR099 strains harboring pXMJ19 *ΔP*_*tac*_ *P*_*tuf*_ *mCherry*, pClik 5α *P*_*tuf*_ *mCherry*, the additional comparator plasmid pCes *P*_*tuf*_ *mCherry*, and the different pClik 5α replication variants (Table 1) were cultivated in a miniaturized microtiter plate system with online monitoring of cell concentration (via optical density) and mCherry fluorescence. Both pClik 5α *P*_*tuf*_ *mCherry* and pXMJ19 Δ*P*_*tac*_ *P*_*tuf*_ *mCherry* were included as standardized controls, enabling comparison of expression outputs across vectors with identical promoter architecture but differing replication systems. The chromosomal reference strain CR099::*P*_*tuf*_ *mCherry*, carrying a single genomic *mCherry* copy, was included to benchmark plasmid-derived expression levels and approximate relative copy number differences (Fig. 1).

Across all constructs, mCherry fluorescence increased in parallel with biomass formation, confirming that the *P*_*tuf*_ promoter drives constitutive expression during exponential growth (Fig. S2, Additional File 1). Plasmid-bearing strains clearly outperformed the chromosomal reference, demonstrating the amplification potential of episomal expression systems in CR099. Among these, pXMJ19 *ΔP*_*tac*_ *P*_*tuf*_ *mCherry* and the unmodified pClik 5α *P*_*tuf*_ *mCherry* plasmids showed moderate, yet robust fluorescence levels, consistent with their established use as standard expression vectors. In contrast, several engineered pClik 5α variants exhibited markedly increased mCherry signals relative to the parental pClik 5α backbone. In particular, constructs carrying the RepA G429E mutation (*repA*^mut^), either alone or in combination with Δ*parAB* or *cgrI*^mut^, showed the strongest fluorescence intensities within the tested set (Fig. 1), indicating substantially elevated plasmid-derived expression. These differences were reproducible across biological replicates (*n* = 2) and were not accompanied by severe growth defects under the applied screening conditions. Comparison with the chromosomal reference revealed that the best-performing pClik 5α variants supported approximately 30–50-fold higher mCherry fluorescence than the single-copy genomic reference, consistent with a strongly increased effective gene dosage. The pCes *P*_*tuf*_ *mCherry* construct showed growth and fluorescence profiles that were not appreciably different from those of the parental pClik 5α *P*_*tuf*_ *mCherry* plasmid under the tested conditions, and we therefore focused subsequent plasmid engineering and physiological analyses on the pClik 5α backbone. Among the pClik 5α variants, pClik 5α *repA*^mut^ *P*_*tuf*_ *mCherry* emerged as a particularly attractive candidate due to its high fluorescence output, while exhibiting reduced growth rates and extended growth phases characteristic of high-copy variants. Thus, the microtiter-plate based mCherry screen enabled rapid discrimination between low-, medium-, and high-expression plasmid variants and identified pClik 5α *repA*^mut^ *P*_*tuf*_ *mCherry* as a lead high-copy vector in *C. glutamicum* CR099 (Fig. 1).

### Plasmid instability of the *repA*^*mut*^*cgrI*^*mut*^ double mutant

Although both the *repA*^*mut*^ and *cgrI*^*mut*^ single mutations increased fluorescence relative to the parental backbone, their combination reduced mCherry production. During screening, colonies of the double-mutant strain displayed visible heterogeneity, with a fraction lacking the characteristic pink coloration. Sequencing of plasmids isolated from three independent non-fluorescent colonies revealed an intact *mCherry* coding sequence but multiple secondary point mutations in *repA* within the ORI region consistent with reduced replication initiation efficiency (Fig. S3, Additional File 1). These counter-selection events are consistent with the interpretation that simultaneous enhancement of replication initiation (*repA*^*mut*^) and derepression of RepA translation (*cgrI*^*mut*^) imposed an excessive replication and expression burden, leading to strong selective pressure for reduced-copy variants. This interpretation is supported by the reduced fluorescence of the double-mutant construct, the occurrence of non-fluorescent colonies, and the presence of compensatory mutations in *repA* while the mCherry cassette remained intact (Fig. S3, Additional File 1). In addition, growth profiles (Fig. S2, Additional File 1) show that strains carrying high-copy plasmids, particularly the *repA*^*mut*^ variant, exhibit reduced growth rates and extended growth phases compared to the parental plasmid, consistent with an increased physiological burden associated with elevated plasmid copy number. Furthermore, reduced and variable fluorescence was observed for the double-mutant construct under non-selective conditions and in minimal medium (Fig. S4, Additional File 1), indicating that plasmid stability is strongly influenced by both replication burden and environmental conditions.

###  Fluorescence-based apparent plasmid dosage estimates identify high-expression variants

To support the inference that increased mCherry fluorescence reflects elevated gene dosage, we derived fluorescence-based apparent plasmid dosage estimates using the chromosomal single-copy mCherry strain CR099::*P*_*tuf*_ *mCherry* as reference. Fluorescence-based comparisons were primarily evaluated during exponential growth, where reporter production is closely coupled to biomass formation, whereas later time points may be influenced by maturation kinetics of mCherry. This approach provides relative comparative estimates rather than absolute plasmid copy numbers and may be influenced by factors such as promoter saturation, and metabolic burden at high expression levels [34, 46, 47]. Therefore, we used this method primarily for comparative ranking and complemented it with independent qPCR-based copy number determination.

For each strain, mCherry fluorescence was plotted against biomass during exponential growth, and the slope of this relationship was determined by linear regression. Representative regression plots and derivations of apparent copy numbers are provided in (Fig. S5, Additional File 1). The ratio of the plasmid-bearing strain slope to the CR099::*P*_*tuf*_ *mCherry* slope served as a relative estimate of mCherry gene dosage and, by extension, plasmid copy number per cell. Using this approach, the parental pClik 5α Ptuf mCherry plasmid exhibited an apparent copy number of approximately 5 copies per cell, consistent with previous reports for low-copy pCG1-family plasmids [30]. The promoter-standardized control pXMJ19 *ΔP*_*tac*_ *P*_*tuf*_ *mCherry* showed clearly higher values, with an estimated about 26 copies per cell, in agreement with its stronger fluorescence output in the screening (Fig. 1).

In contrast, engineered pClik 5α variants carrying the RepA G429E substitution displayed substantially elevated apparent copy numbers relative to the parental backbone. The pClik 5α *repA*^*mut*^ *P*_*tuf*_ *mCherry* plasmid showed the highest apparent gene dosage, with an estimated 48 copies per cell, corresponding to an 8.5-fold increase over the native pClik 5α vector. Variants harboring additional modifications in the *cgrI* promoter or the *parAB* region yielded slightly lower, yet still elevated apparent copy numbers, generally in the range of 16–20 copies per cell, consistent with their intermediate fluorescence intensities in the screening experiment (Fig. 1). Despite carrying several-fold more gene copies than the parental plasmid, strains harboring these high-copy variants maintained stable exponential growth under the tested conditions. Together, the fluorescence-based copy number estimation confirms that the enhanced mCherry expression observed during the screening originates from elevated plasmid dosage in the engineered pClik 5α variants. These findings identified pClik 5α *repA*^*mut*^ as the most promising high-copy system.

### qPCR-based validation confirms elevated plasmid copy number

**Fig. 2:**
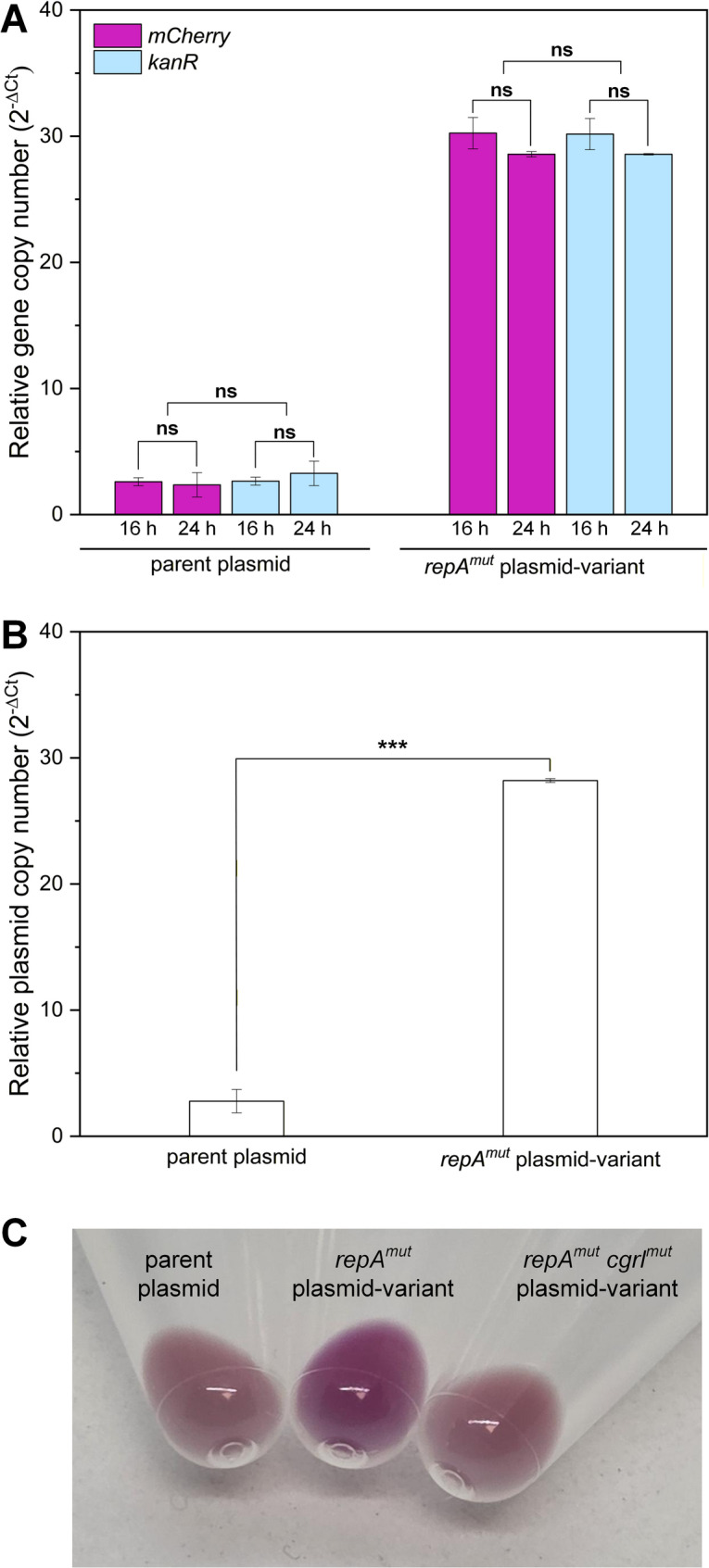
qPCR-based determination of plasmid copy number and phenotypic validation of high-copy variants. (**A**) Relative plasmid copy number of the parental pClik 5α *P*_*tuf*_ *mCherry* plasmid and the high-copy variant pClik 5α *repA*^*mut*^ *P*_*tuf*_ mCherry determined by qPCR using two independent plasmid loci (*mCherry* and *kanR*) normalized to the chromosomal single-copy reference gene *gyrB*. Samples were taken at 16 h and 24 h. No significant differences were observed between target genes or sampling time points (ns, *p* > 0.05). (**B**) Summary of plasmid copy numbers based on combined qPCR data, showing a significantly increased copy number of the *repA*^*mut*^ variant compared to the parental plasmid (***, *p* < 0.001, unpaired two-sided t-test). Values represent means ± SD of biological triplicates (*n* = 3). (**C**) Representative cell pellets obtained under production conditions (GY medium with kanamycin), illustrating increased mCherry accumulation in the *repA*^*mut*^ variant compared to the parental plasmid

To independently validate the fluorescence-based plasmid dosage estimates, we quantified plasmid copy number by quantitative PCR (qPCR) using total DNA and normalization to the chromosomal single-copy reference gene *gyrB* (Fig. 2). Two independent plasmid loci, *mCherry* and *kanR*, were analyzed at 16 h and 24 h to assess robustness across target region and sampling time. The parental plasmid showed low copy numbers in the range of approximately 2–3 copies per chromosome equivalent, whereas the pClik 5α *repA*^*mut*^ *P*_*tuf*_ *mCherry* variant reached approximately 28–30 copies per chromosome equivalent. Thus, the *repA*^*mut*^ variant exhibited an approximately 10-fold higher plasmid copy number than the parental construct (Fig. 2B). Importantly, the qPCR results were highly consistent between the two plasmid targets (*mCherry* and *kanR*) and across both sampling time points, and no significant differences were observed for target gene or sampling time (Fig. 2A). In contrast, the difference between the parental plasmid and the *repA*^*mut*^ variant was highly significant.

Consistent with these quantitative results, visual inspection of cell pellets revealed markedly increased mCherry accumulation in the *repA*^*mut*^ variant compared to the parental plasmid under production conditions (Fig. 2C), providing an intuitive phenotypic confirmation of the elevated plasmid copy number. Together, these qPCR data independently confirm that the markedly increased mCherry expression observed during screening reflects a substantial increase in plasmid copy number, consistent with previous observations that fluorescence-based reporter output correlates well with qPCR-based copy number estimates [48].

### Transcriptome analysis reveals defined and partially shared cellular responses to elevated plasmid load

**Fig. 3:**
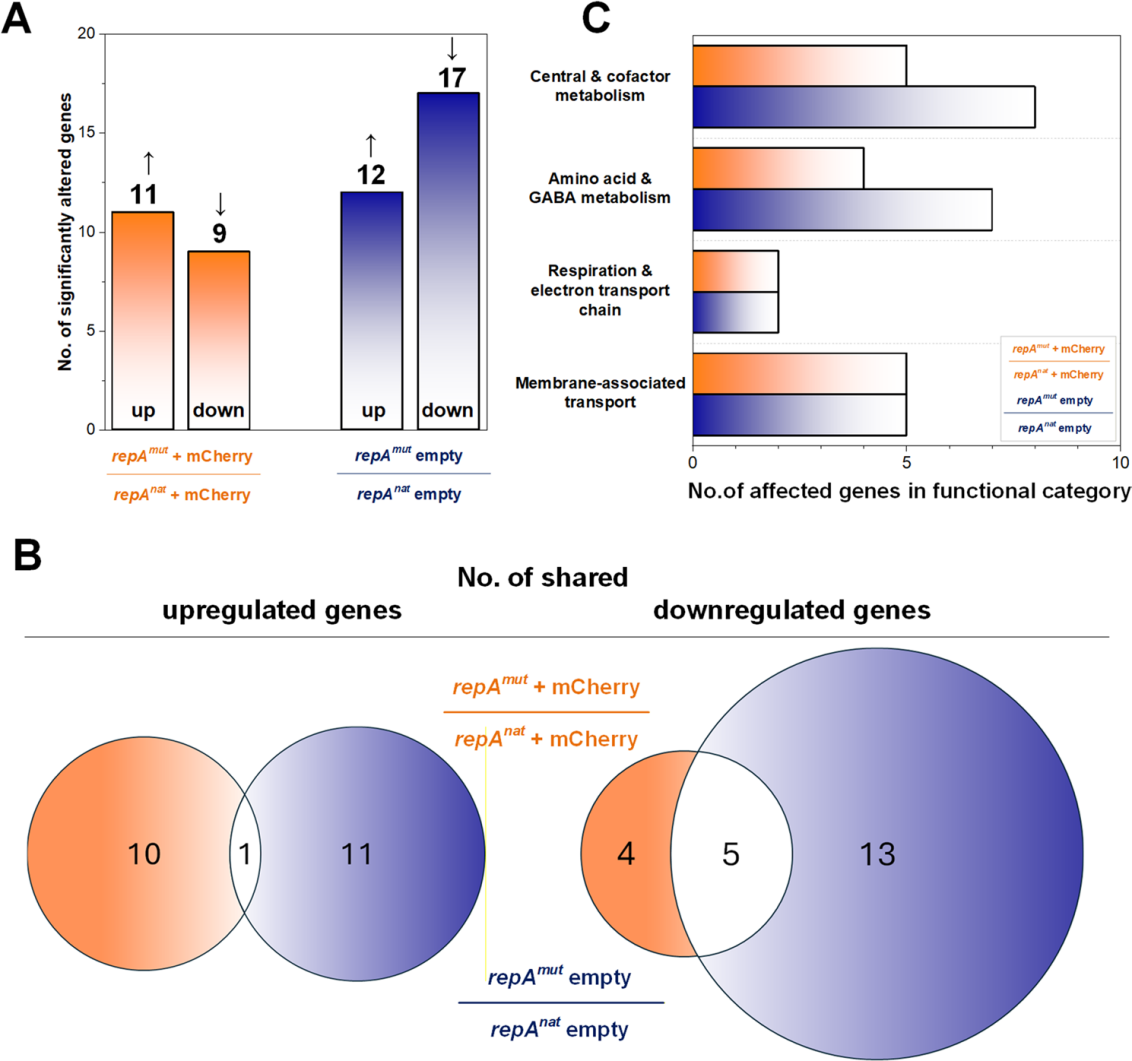
Transcriptome analysis reveals defined and partially shared adaptive responses to increased plasmid copy number. (**A**) Number of significantly up- and downregulated genes (2-Way-ANOVA, *p* < 0.05, absolute fold change ≥ 2) in the comparisons of *repA*^*mut*^ versus native plasmid backgrounds, analyzed in the absence and presence of *mCherry* expression. Data represent biological triplicates (*n* = 3). (**B**) Overlap of significantly regulated genes between the two principal comparisons shown as Venn diagrams for upregulated (left) and downregulated (right) genes. Only genes with consistent regulation direction were considered. (**C**) Distribution of differentially expressed genes across major functional categories based on the same statistical thresholds (2-Way-ANOVA, *p* < 0.05, absolute fold change ≥ 2), highlighting recurring adaptive responses including membrane-associated transport, respiration/electron transport, amino acid and GABA metabolism, and central/cofactor metabolism

To investigate how *C. glutamicum* responds to increased plasmid copy number and to assess the physiological consequences of the high-copy pClik 5α *repA*^*mut*^ replicon, we performed a genome-wide transcriptome analysis. CR099 strains harboring either the native pClik 5α plasmid or its *repA*^*mut*^ derivative, each in the absence or presence of the *mCherry* expression cassette, were cultivated in BHI medium and sampled during mid-exponential growth. Sampling time points and cultivation trajectories are shown in Fig. S6 (Additional File 1). Transcriptome data were analyzed using a stringent statistical framework based on two-way ANOVA (*p* < 0.05, fold-change cutoff of ≥ 2). Analysis focused on two principal comparisons: *repA*^*mut*^ versus native plasmid backgrounds, analyzed both in the absence and presence of *mCherry* expression (Additional File 2). Additional comparisons addressing the transcriptional impact of mCherry expression within each plasmid background were evaluated separately (Additional File 2) and are summarized in Fig. S7 (Additional File 1). Under these criteria, introduction of the *repA*^*mut*^ replicon resulted in a defined set of differentially expressed genes in both conditions (Fig. 3A), and principal component analysis confirmed clear separation of biological replicates and consistent overall transcriptional responses (Fig. S8, Additional File 1).

In the comparison of cells harboring the *repA*^*mut*^ and native empty plasmids, the transcriptional response was broader and included multiple transport-related and membrane-associated functions (Additional File 2). Upregulated genes were predominantly associated with membrane transport and regulatory processes, including diverse transporter families and transcriptional regulators. In contrast, downregulated genes were mainly linked to respiratory functions and amino acid-associated metabolism, including components of the electron transport chain, GABA metabolism, glutamate biosynthesis, and vitamin B6 biosynthesis pathways, as well as siderophore-related transport processes. Overall, this pattern indicates a defined physiological response involving membrane-associated functions, respiratory adjustment, and reorganization of amino acid-related metabolism.

In the comparison of cells harboring the *repA*^*mut*^ and native plasmids in the presence of *mCherry* expression, the transcriptional response was more selective but again involved transport-related and membrane-associated functions. Upregulated genes were associated with transport processes, regulatory functions, and pathways linked to aromatic amino acid and cofactor biosynthesis (Additional File 2). Downregulated genes included functions related to respiration and carbon metabolism, as well as GABA-associated pathways, indicating targeted metabolic adjustments in the expression background. Consistent with this, *mCherry*-associated transcriptional responses showed limited overlap between conditions and were more context-dependent (Fig. S8, Additional File 1), indicating that expression-related effects are secondary to the *repA*-driven response.

Despite these distinct responses, only a small subset of genes showed consistent regulation across both comparisons of the *repA*^*mu*t^ and native plasmid backgrounds, analyzed in the absence and presence of *mCherry* expression (Fig. 3B). Overlap among upregulated genes was minimal, whereas a limited set of downregulated genes was shared between conditions. These included genes related to respiratory functions and amino acid/GABA metabolism, indicating targeted metabolic adjustments rather than a broad, uniform transcriptional response.

**Fig. 4:**
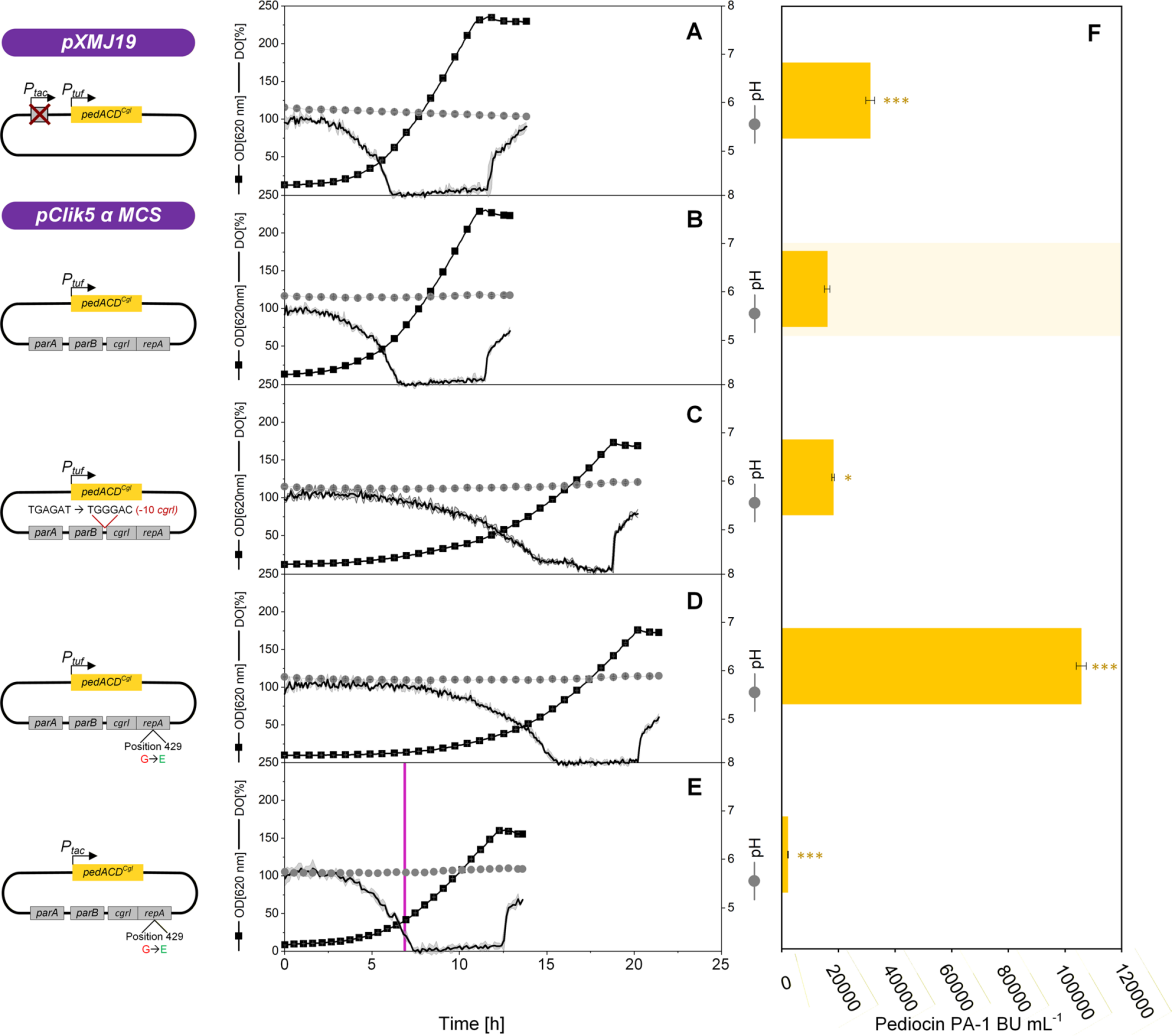
Evaluation of *C. glutamicum* CR099 cell factories expressing the pediocin PA-1 operon (*pedACD*^*Cgl*^). Cultivation experiments were conducted in a miniaturized microtiter plate system under previously established production conditions (700 rpm, 30 °C, GY medium buffered at pH 5.9). The pXMJ19 Δ*P*_*tac*_ *P*_*tuf*_ *pedACD*^*Cgl*^ plasmid lacks the *P*_*tac*_ promoter and the associated *lacI* repressor and enables expression under constitutive *P*_*tuf*_ control. The parental vector pClik 5α *P*_*tuf*_ *pedACD*^*Cgl*^ allows constitutive expression under *P*_*tuf*_ control. Engineered high-copy variants include pClik 5α *cgrI*^*mut*^ *P*_*tuf*_ *pedACD*^*Cgl*^ and pClik 5α *repA*^*mut*^ *P*_*tuf*_ *pedACD*^*Cgl*^, the latter carrying the RepA^G429E^ substitution. For inducible expression, pClik 5α *repA*^*mut*^ *P*_*tac*_ *pedACD*^*Cgl*^ was used, with induction triggered upon dissolved oxygen levels dropping below 10% (purple line). Pediocin PA-1 titers were determined from culture supernatants sampled immediately before oxygen re-entry. Data represent means ± SD of biological triplicates (*n* = 3). Statistical significance was assessed using an unpaired two-sided t-test relative to the parental pClik 5α *P*_*tuf*_ *pedACD*^*Cgl*^ strain and is indicated in the figure (* *p* < 0.05, ** *p* < 0.01, *** *p* < 0.001)

**Fig. 5:**
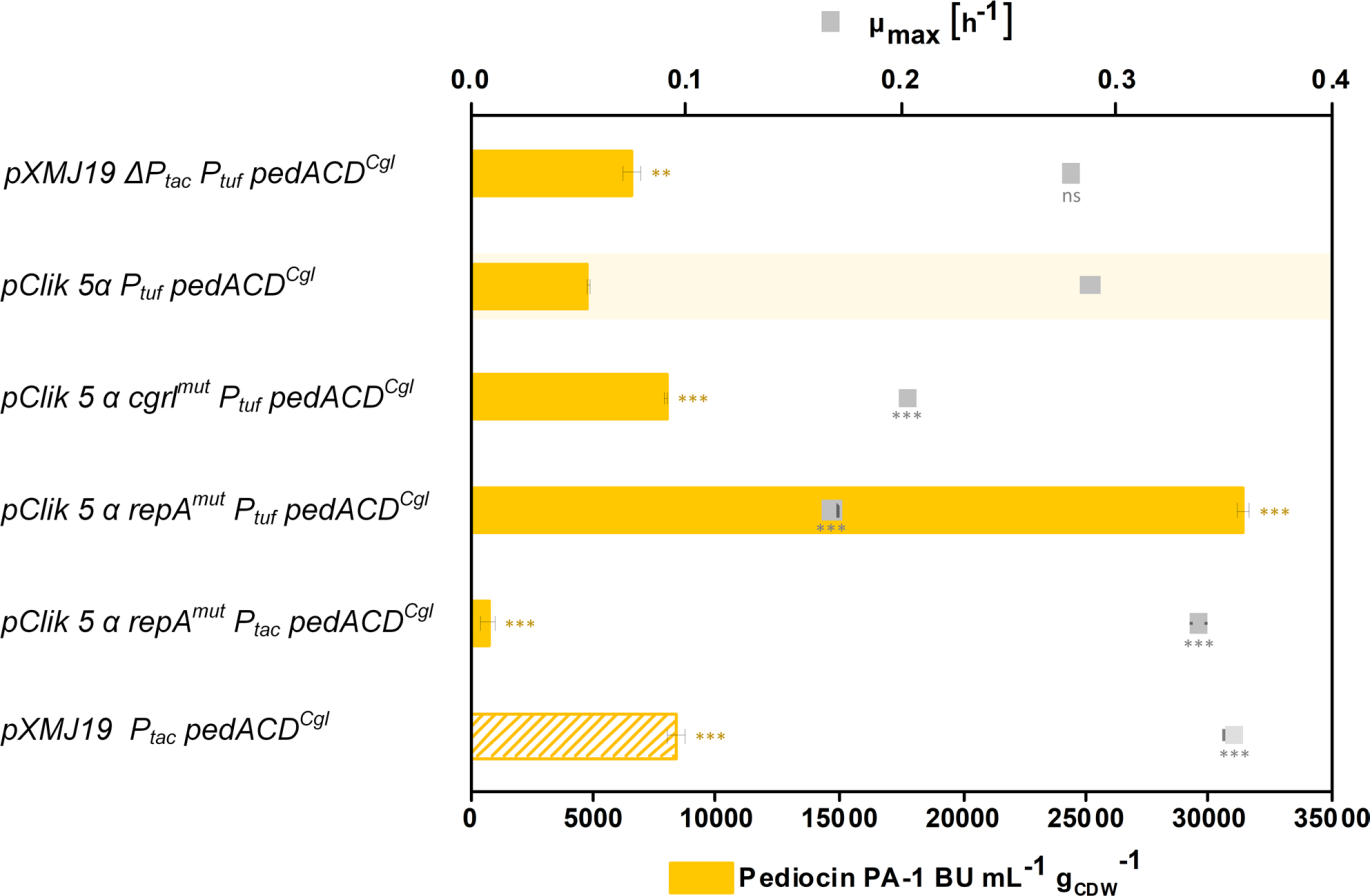
Maximum growth rate and specific production efficiency of strains carrying engineered plasmids. Growth rates and specific production efficiencies were determined during mini-bioreactor cultivations as described for Fig. 4. Specific production efficiency was normalized to the duration of the oxygen-limited phase (required for pediocin PA-1 accumulation) and to the corresponding biomass formation during this period. For benchmarking, the specific production efficiency of *C. glutamicum* CR099 expressing pXMJ19 *P*_*tac*_ *pedACD*^*Cgl*^, calculated from previous medium optimization experiments [86], was included. Data represent means ± SD of biological triplicates (*n* = 3). Statistical significance was assessed using an unpaired two-sided t-test relative to the parental pClik 5α *Ptuf* *pedACD*^*Cgl*^ strain and is indicated in the figure (* *p* < 0.05, ** *p* < 0.01, *** *p* < 0.001)

At the functional level, several recurring response themes were nevertheless apparent across the analyzed comparisons (Fig. 3C), most prominently membrane-associated transport processes, respiratory functions, and amino acid-related metabolism. Thus, the transcriptome data point to a structured adaptive response to elevated plasmid copy number, characterized by selective physiological adjustments rather than global transcriptional collapse. Having established that the high-copy replicon induces defined and manageable transcriptional changes, we next evaluated its impact on recombinant pediocin PA-1 production under controlled cultivation conditions (Figs. 4 and 5).

###  High-copy pClik 5α variants enhance pediocin PA-1 production

To assess the impact of the engineered pClik 5α variants on peptide production, we evaluated their performance for the synthesis of pediocin PA-1. CR099 strains harboring the codon-optimized *pedACD*^*Cgl*^ operon under control of either the constitutive *P*_*tuf*_ promoter or the inducible *P*_*tac*_ promoter were cultivated in a miniaturized bioreactor system under previously optimized process conditions (GY medium buffered at pH 5.9, 2 g L^− 1^ CaCl_2_, oxygen-limited production phase) [38] (Fig. 4).

Introduction of the high-copy pClik 5α *repA*^mut^ backbone resulted in a reproducible increase in pediocin production. Under constitutive *P*_*tuf*_ control, the pClik 5α *repA*^mut^ *P*_*tuf*_ *pedACD*^*Cgl*^ strain achieved the highest titers within this study under identical cultivation conditions, clearly outperforming the parental pClik 5α system and the pXMJ19-based reference (Fig. 4). Variants carrying the *cgrI*^mut^ modification showed a moderate increase in productivity, while constructs combining *repA*^mut^ with Δ*parAB* or *cgrI*^mut^ exhibited intermediate phenotypes, consistent with their slightly reduced plasmid copy numbers. To assess the influence of expression timing on productivity, we additionally evaluated strains carrying the pClik 5α *repA*^mut^ *P*_*tac*_ *pedACD*^*Cgl*^ plasmid, where pediocin expression was induced upon detection of oxygen depletion (< 10% dissolved oxygen). These strains produced lower titers than their constitutive counterparts, reflecting differences in expression timing and cumulative production rather than intrinsic plasmid performance. Differences between plasmid systems should therefore be interpreted within the context of the respective expression mode and cultivation conditions.

Growth rate and specific production efficiency were calculated for each strain (Fig. 5). While strains harboring high-copy plasmids exhibited slightly reduced maximum growth rates, their specific production efficiencies during the oxygen-limited production phase were markedly improved. In particular, the pClik 5α *repA*^mut^ *P*_*tuf*_ *pedACD*^*Cgl*^ strain achieved the highest efficiency among all constructs tested, outperforming both pXMJ19-based systems and previously optimized pH- and Ca^2+^-controlled processes under the applied mini-bioreactor conditions (Fig. 5).

### Benchmarking of high-copy pClik 5α variants against previously reported pediocin PA-1 production systems

**Fig. 6:**
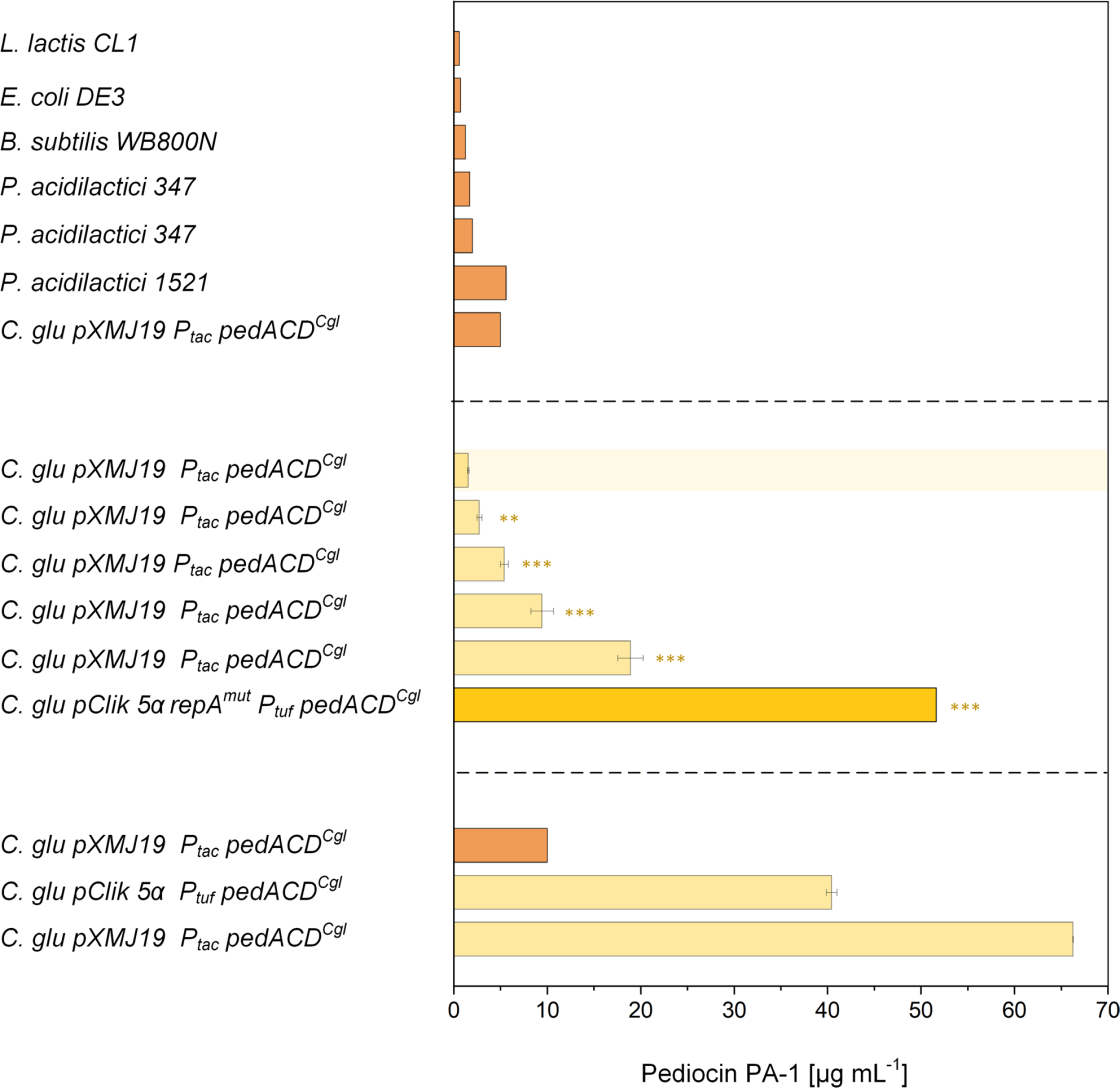
Benchmarking of pediocin PA-1 production using high-copy plasmid systems. Pediocin PA-1 titers obtained with *C. glutamicum* CR099 expressing pClik 5α *repA*^*mut*^*P*_*tuf*_*pedACD*^*Cgl*^ are compared to previously reported production systems. The upper panel summarizes shake-flask and test-tube data from literature sources, including *Lactococcus lactis* CL1 [18], *Bacillus subtilis* [16], *Escherichia coli* producing the M31L peptide variant [11], *Pediococcus acidilactici* 347 [17], *C. glutamicum* CR099 pXMJ19 Ptac pedACDCgl [19], and *P. acidilactici* 1521 [98]. The middle panel shows results from process optimization experiments reported previously [86]. The highlighted bar indicates the pediocin PA-1 titers achieved in this study using the high-copy plasmid pClik 5α *repA*^*mut*^*P*_*tuf*_*pedACD*^*Cgl*^ under optimized mini-bioreactor conditions (700 rpm, 30 °C, GY medium, pH 5.9, 2 g L⁻¹ CaCl_2_). The lower panel summarizes pediocin PA-1 production in bioreactor processes reported previously [86], including both fed-batch and batch cultivations under optimized conditions. Data generated in this study represent means ± SD of biological triplicates (*n* = 3). Statistical significance is shown for the middle panel (data from [86]) and for data generated in this study (highlighted bar) and was assessed using an unpaired two-sided t-test and is indicated in the figure (* *p* < 0.05, ** *p* < 0.01, *** *p* < 0.001)

To place the performance of the engineered high-copy plasmid system into context, we compared the pediocin PA-1 titers obtained with the pClik 5α *repA*^mut^ *P*_*tuf*_ *pedACD*^*Cgl*^ strain to values reported in the literature for different microbial hosts and cultivation formats (Fig. 6). Published production systems include *Lactococcus lactis* CL1, *Pediococcus acidilactici* strains 347 and 1521, *Bacillus subtilis*, heterologous *E. coli* producers of the M31L variant, and earlier *C. glutamicum* processes based on pXMJ19-derived vectors (Fig. 6, upper and middle frames). Reported pediocin titers for these systems typically fall within a modest range. Native and heterologous LAB and *B. subtilis* producers commonly achieve 5–20 mg L^− 1^, while *E. coli* systems generally reach ≤ 10 mg L^− 1^ under shake-flask conditions. Previous *C. glutamicum* processes operated under optimized microaerobic and acidic conditions with Ca²⁺ supplementation yielded 30–40 mg L^− 1^ in mini-bioreactors.

Under identical mini-bioreactor conditions, the pClik 5α *repA*^mut^ *P*_*tuf*_ *pedACD*^*Cgl*^ construct reached pediocin PA-1 titers that were approximately 2.5-fold higher than those obtained with the best-performing pXMJ19-based control strain used previously (Fig. 6, middle frame). When compared to literature values, the measured titer lies within or above the upper range reported for other microbial hosts, both native and heterologous, when cultivated under comparable conditions. Taken together, these benchmarking results show that the high-copy pClik 5α *repA*^mut^ system achieved the highest pediocin PA-1 titers among all systems evaluated in this study and, to our knowledge, falls within the higher range reported for comparable systems under similar cultivation setups.

## Discussion

###  High-copy plasmid engineering unlocks new expression space in pCG1-family vectors

This study establishes a rational and systematic route to elevate plasmid copy number in *C. glutamicum* through targeted modification of the pCG1-family replication-origin architecture. Our fluorescence-based screening clearly revealed substantial differences in gene dosage between commonly used plasmid backbones: the pBL1-derived pXMJ19 replicated at higher apparent copy numbers than the parental pClik 5α vector, consistent with earlier observations that pXMJ19 frequently enables superior production performance [38]. These findings underscore that replication origin architecture is a major, and often underexplored, determinant of heterologous expression capacity in *C. glutamicum*. In this context, the comparison between pCG1- and pBL1-family plasmids highlights how replication mechanism influences plasmid behavior, with rolling-circle replicons generally supporting higher copy numbers at the expense of stability, whereas theta-type replicons provide more stable maintenance at lower copy numbers [28, 35]. Recent advances in *C. glutamicum* expression vector design, including improved plasmid backbones such as pBKEx2 and pSJEx3, further highlight the importance of vector architecture for optimizing recombinant protein production and complement strategies based on replication-origin engineering [49, 50]. Beyond their role as expression vectors, these systems have been widely applied as versatile genetic backbones for metabolic engineering in *C. glutamicum*. In particular, pClik-based platforms, together with closely related pCG1-family vectors such as pCes, have enabled the stable expression of heterologous enzymes and multi-gene pathways for substrate uptake engineering, metabolic flux redirection, and the production of value-added compounds [39, 51, 52]. These applications highlight the robustness and modularity of the pClik platform across diverse engineering contexts, supporting reliable gene expression under both screening and process-relevant conditions.

Despite the central role of replication origins, few studies have systematically mapped how individual components of pCG1-family replicons contribute to tunable copy-number ranges. In this work, independent qPCR-based plasmid copy number determination confirmed that the engineered pClik 5α *repA*^*mut*^ variant operates in a substantially higher copy-number regime (approximately 28–30 copies per chromosome equivalent) compared to the parental plasmid (approximately 2–3 copies). These data validate the fluorescence-based screening approach and establish the *repA*^*mut*^ replicon as a high-copy variant within the pCG1-family framework.

Within the pClik 5α backbone, both mechanistically informed strategies - antisense RNA modulation (*cgrI* promoter mutation) and enhanced replication initiation (RepA G429E) - increased copy number, with the RepA variant delivering the most pronounced effect among the tested constructs. These outcomes are consistent with proposed mechanisms, namely that reduced *cgrI* transcription increases *repA* translation [53] and thereby controls plasmid copy number, as demonstrated previously for pCG1-family replicons [30], while RepA G429E likely strengthens ORI binding or initiation frequency [29]. Notably, combining both mutations destabilized plasmid maintenance and reproducibly triggered loss-of-function mutations in *repA*, defining a clear physiological ceiling for pCG1-family replication, where fluorescence output decreased and colonies frequently segregated into non-fluorescent phenotypes. Sequencing revealed multiple compensatory mutations in *repA* while leaving the *mCherry* cassette intact (Fig. S3, Additional File 1). These results are compatible with an over-initiation toxicity scenario, in which excessive replication load exceeds the cellular capacity to maintain high copy numbers.

Analysis of upstream ORFs provided additional mechanistic insight. In the present study, *parA* and *parB* were deleted simultaneously, and therefore the observed effects reflect the removal of the partitioning module as a whole rather than individual gene contributions. Their deletion markedly decreased copy number, and sequence similarity to the *per* gene of pGA1 suggests a stabilizing maintenance function that has not previously been described for the pClik 5α backbone [54]. Previous work on pCG1-family plasmids indicates that *parB* may play a dominant role in copy number control, whereas *parA* appears to have a more limited contribution [30]. Consequently, the individual contributions of these genes cannot be resolved from the current dataset, and targeted dissection of *parA* and *parB* separately represents an important direction for future studies. Together, these observations raise the possibility that the pClik 5α plasmid carries a per-like maintenance function and highlight a previously underappreciated stabilizing role within the pCG1 backbone, although this interpretation remains putative and requires further validation.

Overall, these insights delineate the accessible engineering space of pCG1-family replicons and identify actionable levers for future vector optimization. The systematically engineered high-copy pClik 5α variants thus provide a promising platform for controlled tuning of gene dosage in *C. glutamicum*. Our observations further suggest that plasmid stability in high-copy systems is not solely determined by intrinsic replication control but is also strongly modulated by environmental conditions such as nutrient availability and selection pressure.

###  Transcriptome signatures reveal defined and adaptive responses to elevated plasmid copy number

Increasing plasmid load is frequently associated with metabolic and physiological burden in bacterial hosts [46, 55, 56]. In the present study, transcriptome analysis based on a stringent statistical framework revealed a defined and limited set of differentially expressed genes in response to the high-copy pClik 5α *repA*^*mut*^ replicon. Rather than indicating a global disruption of cellular homeostasis, the observed transcriptional changes point to targeted and adaptive adjustments, consistent with previous systems-level analyses of recombinant protein production in bacteria [55, 57–59].

At the level of individual genes, overlap between the two principal comparisons was limited, indicating that transcriptional responses depend in part on the expression context. However, recurring functional patterns were observed across both conditions. In particular, genes associated with membrane-associated transport, respiratory functions, and amino acid-related metabolism were consistently affected. Notably, components of the electron transport chain (e.g., cytochrome oxidase subunits) and genes involved in GABA and amino acid metabolism were among the shared downregulated targets, suggesting coordinated adjustments in energy metabolism and nutrient utilization, as also observed in other bacterial systems under increased biosynthetic demand [55, 56].

Beyond these shared elements, comparison-specific responses further highlight the adaptive nature of the system. In the absence of heterologous expression, the response to increased plasmid copy number included a broader set of changes involving transport processes, regulatory functions, and metabolic pathways. In contrast, in the presence of *mCherry* expression, the response was more selective and reflected additional adjustments linked to protein production and associated metabolic demands. These differences indicate that the cellular response to elevated plasmid copy number is modulated by the presence of recombinant protein expression, consistent with previous observations linking gene dosage, expression burden, and metabolic adaptation [55, 57].

Importantly, despite these transcriptional adjustments, no evidence for widespread downregulation of essential biosynthetic pathways or collapse of central metabolism was observed. Instead, the data support a model in which *C. glutamicum* accommodates increased plasmid copy number through selective modulation of transport, respiration, and metabolic functions. This structured and adaptive response contrasts with observations in other bacterial systems, where high plasmid loads often result in severe growth defects or instability [46, 56], and underscores the suitability of *C. glutamicum* as a robust chassis for high-copy expression systems [20–22].

###  High-copy pClik 5α variants unlock markedly higher pediocin PA-1 titers and point to remaining bottlenecks in peptide maturation

Plasmid-based expression has long been central to the *C. glutamicum* biotechnology toolbox, with pBL1- and pCG1-derived vectors, promoter libraries, and integrative systems enabling finely tuned expression of native and heterologous genes [34, 37, 60–64]. In this study, constitutive expression systems were deliberately employed to directly assess the effect of plasmid copy number on gene dosage and expression performance, independent of additional regulatory layers such as inducible control systems. Likewise, substantial prior work in lactic acid bacteria, *Bacillus subtilis*, and *E. coli* has mapped biosynthetic and secretion requirements for pediocin PA-1 and underscored the challenges of attaining commercially relevant yields [15–18, 65]. Our study extends these foundations by systematically applying replication-origin engineering to a widely used pCG1-family vector and integrating high-throughput reporter screening and transcriptomics to define the accessible copy-number range and its physiological consequences. The engineered pClik 5α variants generated here—especially the RepA^G429E^ replicon - help narrow the performance gap to high-expressing pBL1-family vectors while preserving stable maintenance, thereby expanding the genetic toolbox available for *C. glutamicum* strain development. Under previously optimized process conditions, the pClik 5α *repA*^*mut*^ vector increased titers by 2.5-fold over the best earlier system [19, 38], positioning *C. glutamicum* within the higher end of ranges reported for LAB, *Bacillus subtilis*, and *E. coli* producers under comparable cultivation formats. These findings indicate that increased plasmid copy number acts as a primary driver of enhanced production, but that overall system performance is additionally shaped by the balance between expression strength, cellular capacity, and downstream processes such as protein folding and maturation. This combined effect addresses a key upstream bottleneck that has historically hindered commercialization of pediocin [66].

However, the data also illustrate the operational ceiling of episomal pediocin production. Expression strength and plasmid copy number are closely linked, as high transcriptional activity increases cellular burden and can influence plasmid stability, highlighting the need to balance both parameters for optimal performance. Increasing gene dosage via high-copy plasmids improved titers but reduced growth rate and imposed replicative stress, particularly when combined with additional *cgrI* mutations. These limitations likely reflect not only transcription–translation load but also bottlenecks intrinsic to pediocin biosynthesis. Importantly, pediocin PA-1 titers in this study were quantified using an activity-based inhibition assay, which selectively reports the fraction of biologically active peptide. Consequently, the total amount of pediocin produced—including misfolded species, incorrectly bridged intermediates, or conformationally inactive variants—cannot be resolved with this method. Pediocin PA-1 folding requires the correct formation of two disulfide bonds that stabilize the peptide scaffold [67]. Heterologous cytosolic expression can therefore yield misfolded or partially bridged peptides due to non-native redox conditions, which may contribute to folding challenges and the observed proteostatic responses [68, 69].

The increased production demand likely imposes a folding-related burden, which may represent an additional limiting factor for production performance beyond plasmid copy number alone, as also suggested in other recombinant protein production systems. Several strategies may thus complement high-copy plasmid engineering. In silico modeling suggests that introduction of a third disulfide bond could enhance structural stability [70], an approach that could be experimentally explored in *C. glutamicum*. Analogous to DsbC co-expression and reductive-pathway attenuation in *E. coli* [71], enhancing folding pathways in *C. glutamicum*—via CdbC overexpression [72] or modulation of mycothiol/thioredoxin systems [73]—may promote correct disulfide bond formation. Because of the tight coupling between redox and protein synthesis and folding [74], targeted manipulation of cytosolic redox balance, which has been applied in *C. glutamicum* for other biotechnological processes, could potentially support improved folding, though this remains to be demonstrated for pediocin [40, 75, 76].

Altogether, these data indicate that high-copy plasmid engineering is an effective strategy to enhance AMP productivity. Further improvements may be achieved by combining this approach with folding, redox, and secretion engineering to reach maximal performance. The pClik 5α *repA*^*mut*^ backbone generated here provides a robust basis for further development of *C. glutamicum* producers of structurally demanding peptides.

The present study demonstrates the utility of this system using two representative expression cases—mCherry as an intracellular reporter and pediocin PA-1 as a secreted antimicrobial peptide. Further validation with additional protein classes, including larger enzymes and alternative secretion targets, will be important to define the full application scope of the platform.

## Conclusions

In this study, we developed and systematically assessed a family of high-copy plasmids designed to enhance recombinant protein and antimicrobial peptide production in *C. glutamicum*. Targeted modification of the pClik 5α replication-control region yielded variants with substantially elevated plasmid copy numbers and correspondingly increased transcriptional output, resulting in enhanced heterologous expression capacity. The RepA^G429E^ variant emerged as the most effective replicon, supporting the highest plasmid abundance, strongest mCherry reporter output, and, when applied to pediocin PA-1 biosynthesis, the best production performance.

Genome-wide transcriptomic profiling revealed that elevated plasmid and expression load was associated with defined transcriptional adjustments involving membrane-associated transport, respiratory functions, and amino acid-related metabolism. These responses underscore the intrinsic robustness of *C. glutamicum* to withstand elevated replication and folding demands and highlight the suitability of the microbe as a chassis for high-level production of structurally complex peptides.

Application of the optimized high-copy plasmid pClik 5α *repA*^*mut*^ *P*_*tuf*_ *pedACD*^*Cgl*^ yielded pediocin PA-1 titers approximately 2.5-fold higher than the best-performing pXMJ19-based system under otherwise identical cultivation conditions, placing this platform, to our knowledge, within the upper range of reported microbial processes for pediocin PA-1 production under comparable conditions. These gains were achieved without process re-optimization, indicating that tuning plasmid replication alone can yield substantial improvements in heterologous peptide biosynthesis.

Together, this work establishes high-copy pClik 5α derivatives as a promising high-copy expression platform for *C. glutamicum*. Beyond pediocin PA-1, these plasmids might broaden the accessible expression space for structurally complex peptides and recombinant proteins, which should be explored in future studies. Future efforts integrating plasmid copy-number control with targeted engineering of folding catalysts, disulfide-bond isomerases such as CdbC, and redox-balancing pathways may further expand achievable yields. In this regard, high-copy plasmid engineering, when combined with host and process optimization, might offer a generalizable route toward robust *C. glutamicum* cell factories for industrially relevant bioactive peptides.

## Materials and methods

### Microorganisms, plasmids, and genes

*C. glutamicum* CR099, a genome-reduced strain, based on the wildtype ATCC 13,032 [77], as well as the pediocin PA-1 sensor strains *Listeria innocua LMG2785 pIMK2 (kan*^*R*^*) and LMG2785 pNZ44 (cm*^*R*^*)* were obtained from previous work [19]. *E. coli* DH10B and NM522 (Invitrogen, Carlsbad, CA, USA) were used as hosts for plasmid amplification and methylation, respectively.

The plasmids pClik 5α [78] and pXMJ19 [79] were used for episomal overexpression in *C. glutamicum*. The episomal vectors used in this study carry origins of replication for *E. coli* (ORI^Ec^) and *C. glutamicum* (ORI^Cgl^). The *C. glutamicum* replication modules belong to either the pCG1 family (pClik 5α, pCes), which replicate via a rolling-circle mechanism, or the pBL1 family (pXMJ19), which replicate via a theta-type mechanism. The vectors confer kanamycin resistance (pClik 5α, pCes) or chloramphenicol resistance (pXMJ19), respectively. The vector pCes, a pCG1-family shuttle plasmid carrying ORI^Cgl^ and ORI^Ec^ and conferring kanamycin resistance, was included only in the initial screening [80] (Fig. 1). The integrative plasmid pClik 5α int sacB was employed for genome-based modifications [81]. It comprises a kanamycin resistance marker (kan^R^), the *sacB* gene from *Bacillus subtilis* encoding levansucrase, a multiple cloning site (MCS), and the ORI^Ec^ [82, 83]. Plasmid DNA was methylated by co-expression of plasmid pTC in *E. coli* NM522. This plasmid carries the *C. glutamicum* methyltransferase, an ORI^Ec^ locus, and a tetracycline resistance marker [84]. The pediocin PA-1 gene cluster (*pedACDCgl*), codon-optimized for *C. glutamicum*, was obtained from previous work [19]. The native sequence of the *mCherry* gene was used for plasmid copy number estimation [85]. All strains and plasmids used in this study are listed in Table 1. The sequence of the plasmid pClik 5α MCS is provided in Table S1 (Additional File 1). Primer sequences are provided in Table S2 (Additional File 1).

**Table 1:** Strains and plasmids used in this study

Strain	Description	Reference
* E. coli *		
DH10B	Cloning host	[95]
NM522	Cloning host	Invitrogen
*L. innocua*		
pIMK2	Sensor strain expressing the plasmid pIMK2 *kan*^*R*^	[19]
pNZ44	Sensor strain expressing the plasmid pNZ44 *cm*^*R*^	[19]
*C. glutamicum*		
CR099	Genome-reduced derivative of strain ATCC 13,032 with deletion of *ΔCGP1-3* and *ΔISCg1-2*	[43]
CR099 pXMJ19 *P*_*tac*_ *pedACD*^*Cgl*^	CR099 with episomal expression of the codon-optimized pediocin operon from *P. acidilactici* under the control of *P*_*tac*_	[19]
CR099 pXMJ19 Δ*P*_*tac*_ *P*_*tuf*_ *mCherry*	CR099, expressing the episomal plasmid pXMJ19 ΔP_tac_ with the *mCherry*-gene under the control of *P*_*tuf*_	This work
CR099 pXMJ19 Δ*P*_*tac*_ *P*_*tuf*_ *pedACD*^*Cgl*^	CR099, expressing the episomal plasmid pXMJ19 Δ*P*_*tac*_ with the codon-optimized pediocin PA-1 operon derived from *P. acidilactici* under the control of *P*_*tuf*_	This work
CR099 pClik 5α *P*_*tuf*_ *mCherry*	CR099, expressing the episomal plasmid pClik *5α* with the *mCherry*-gene under the control of *P*_*tuf*_	This work
CR099 pClik 5α *repA*^*mut*^ *P*_*tuf*_ *mCherry*	CR099, expressing the episomal plasmid pClik *5α*, harboring the mutated RepA^G429E^ variant, and the *mCherry*-gene under the control of *P*_*tuf*_	This work
CR099 pClik 5α *cgrI*^*mut*^ *P*_*tuf*_ *mCherry*	CR099, expressing the episomal plasmid pClik 5α, harboring a mutation within the promoter of the *cgrI* antisense-RNA, and the *mCherry*-gene under the control of *P*_*tuf*_	This work
CR099 *pClik 5α repA*^*mut*^ *cgrI*^*mut*^ *P*_*tuf*_ *mCherry*	CR099, expressing the episomal plasmid pClik 5α, harboring the mutated RepA^G429E^ variant, a mutation within the promoter of the *cgrI* antisense-RNA, and the *mCherry*-gene under the control of *P*_*tuf*_	This work
CR099 pClik 5α Δ*parAB P*_*tuf*_ *mCherry*	CR099, expressing the episomal plasmid pClik 5α, which features the deletion of potential partitioning genes (Δ*parAB*), and the *mCherry*-gene under the control of *P*_*tuf*_	This work
CR099 pClik 5α Δ*parAB* *repA*^*mut*^ *P*_*tuf*_ *mCherry*	CR099, expressing the episomal plasmid pClik 5α, which features the deletion of potential partitioning genes (Δ*parAB*), the mutated RepA^G429E^ variant, and the *mCherry*-gene under the control of *P*_*tuf*_	This work
CR099 pClik 5α Δ*parAB* *cgrI*^*mut*^ *P*_*tuf*_ *mCherry*	CR099, expressing the episomal plasmid pClik 5α, which features the deletion of potential partitioning genes (Δ*parAB*), a mutation within the promoter of the *cgrI* antisense-RNA, and the *mCherry*-gene under the control of *P*_*tuf*_	This work
CR099 pClik 5α Δ*parAB cgrI*^*mut*^ *repA*^*mut*^ *P*_*tuf*_ *mCherry*	CR099, expressing the episomal plasmid pClik 5α, which features the deletion of potential partitioning genes (Δ*parAB*), a mutation within the promoter of the *cgrI* antisense-RNA, the mutated RepA^G429E^ variant, and the *mCherry*-gene under the control of *P*_*tuf*_	This work
CR099 pCes *P*_*tuf*_ *mCherry*	CR099, expressing the episomal plasmid pCes with the *mCherry*-gene under the control of *P*_*tuf*_	This work
CR099 pClik 5α *P*_*tuf*_ *pedACD*^*Cgl*^	CR099 with episomal expression of the codon-optimized pediocin operon from *P. acidilactici* under control of *P*_*tuf*_	[86]
CR099 pClik 5α *repA*^*mut*^ *P*_*tuf*_ *pedACD*^Cgl^	CR099, expressing the episomal plasmid pClik 5α, including the mutation RepA^G429E^, with the codon-optimized pediocin PA-1 operon derived from *P. acidilactici* under the control of *P*_*tuf*_	This work
CR099 pClik 5α *cgrI*^*mut*^ *P*_*tuf*_ *pedACD*^*Cgl*^	CR099, expressing the episomal plasmid pClik 5α, including a mutation within the promoter region of the antisense-RNA *cgrI*, and the codon-optimized pediocin PA-1 operon derived from *P. acidilactici* under the control of *P*_*tuf*_	This work
CR099 pClik 5α *repA*^mut^ *P*_*tac*_ *pedACD*^Cgl^	CR099, expressing the episomal plasmid pClik 5α, harboring the mutated RepA^G429E^ variant, and the codon-optimized pediocin PA-1 operon derived from *P. acidilactici* under the control of *P*_*tac*_ [30]	This work
CR099::*P*_*tuf*_ *mCherry*	Strain with genome-based expression of the *mCherry*-gene under the control of *P*_*tuf*_	This work
*Plasmids*		
pClik 5α MCS	Episomal vector, *ORI*^*Cgl*^, *ORI*^*Ec*^, k*an*^*R*^	[96]
pXMJ19	Episomal vector, *P*_*tacI*_ *lacI*, *ORI*^*Cgl*^, *ORI*^*Ec*^, *cm*^*R*^	[19]
pCes	Episomal vector, *ORI*^*Cgl*^, *ORI*^*Ec*^, k*an*^*R*^	[97]
pXMJ19 Δ*P*_*tac*_	Shuttle vector derived from pxMJ19, *ORI*^*Ec*^, *ORI*^*Cgl*^, *cm*^*R*^, Δ*P*_*tacI*_, Δ*lacI*	This work
pXMJ19 Δ*P*_*tac*_ *P*_*tuf*_ *mCherry*	Episomal expression of the *mCherry*-gene under the control of *P*_*tuf*_	This work
pXMJ19 Δ*P*_*tac*_ *P*_*tuf*_ *pedACD*^*Cgl*^	Episomal expression of the codon-optimized pediocin PA-1 operon derived from *P. acidilactici* under the control of *P*_*tuf*_	This work
pClik 5α MCS	Episomal vector, *ORI*^*Cgl*^, *ORI* ^*Ec*^, *kan*^*R*^	[78]
pClik 5α *P*_*tuf*_ *mCherry*	Episomal expression of the *mCherry*-gene under the control of *P*_*tuf*_	This work
pClik 5α *repA*^*mut*^ *P*_*tuf*_ *mCherry*	Episomal expression of the *mCherry*-gene under the control of *P*_*tuf*_, based on pClik 5α harboring the mutated RepA^G429E^ variant	This work
pClik 5α *cgrI*^*mut*^ *P*_*tuf*_ *mCherry*	Episomal expression of the *mCherry*-gene under the control of *P*_*tuf*_, based on pClik 5α harboring a mutation within the promoter of the *cgrI* antisense-RNA	This work
pClik 5α *repA*^*mut*^ *cgrI*^*mut*^ *P*_*tuf*_ *mCherry*	Episomal expression of the *mCherry*-gene under the control of *P*_*tuf*_, based on pClik 5α harboring the mutated RepA^G429E^ variant and a mutation within the promoter of the *cgrI* antisense-RNA	This work
pClik 5α Δ*parAB P*_*tuf*_ *mCherry*	Episomal expression of the *mCherry*-gene under the control of *P*_*tuf*_, based on pClik 5α which features the deletion of the potential partitioning genes (Δ*parAB*)	This work
pClik 5α Δ*parAB repA*^*mut*^ *P*_*tuf*_ *mCherry*	Episomal expression of the *mCherry*-gene under the control of *P*_*tuf*_, based on pClik 5α which features the deletion of the potential partitioning genes (Δ*parAB*) and the mutated RepA^G429E^ variant	This work
pClik 5α Δ*parAB cgrI*^*mut*^ *P*_*tuf*_ *mCherry*	Episomal expression of the *mCherry*-gene under the control of *P*_*tuf*_, based on pClik 5α which features the deletion of the potential partitioning genes (Δ*parAB*) and a mutation within the promoter of the *cgrI* antisense-RNA	This work
pClik 5α Δ*parAB cgrI*^*mut*^ *repA*^*mut*^ *P*_*tuf*_ *mCherry*	Episomal expression of the *mCherry*-gene under the control of *P*_*tuf*_, based on pClik 5α which features the deletion of the potential partitioning genes (Δ*parAB*), a mutated RepA^G429E^ variant and a mutation within the promoter of the *cgrI* antisense-RNA	This work
pClik 5α int sacB *P*_*tuf*_ *mCherry*	Vector for genome integration of the *mCherry*-gene under control of *P*_*tuf*_ into *bioD* (*NCgl*2516)	This work
pClik 5α *P*_*tuf*_ *pedACD*^Cgl^	Episomal expression of the codon-optimized pediocin PA-1 operon derived from *P. acidilactici* under the control of *P*_*tuf*_	This work
pClik 5α *repA*^*mut*^ *P*_*tuf*_ *pedACD*^*Cgl*^	Episomal expression of the codon-optimized pediocin PA-1 operon derived from *P. acidilactici* under the control of *P*_*tuf*_, based on the plasmid pClik *5α* harboring the mutated RepA^G429E^ variant	This work
pClik 5α *cgrI*^*mut*^ *P*_*tuf*_ *pedACD*^*Cgl*^	Episomal expression of the codon-optimized pediocin PA-1 operon derived from *P. acidilactici* under the control of *P*_*tuf*_, based on the plasmid pClik 5α harboring a mutation within the promoter of the *cgrl* antisense-RNA	This work
pClik 5α *repA*^*mut*^ *P*_*tac*_ *pedACD*^*Cgl*^	Episomal expression of the codon-optimized pediocin PA-1 operon derived from *P. acidilactici* under the control of *P*_*tac*_, based on the plasmid pClik 5α harboring the mutated RepA^G429E^ variant	This work
pCes Δ*P*_*tac*_ *P*_*tuf*_ *mCherry*	Episomal expression of the *mCherry*-gene under the control of *P*_*tuf*_	This work

The sequence of the plasmid pClik 5α MCS is provided in GenBank format in the Additional File 1

###  Growth media

Pre-cultures and cultures for genome-wide transcriptome analysis were conducted in BHI medium (37 g L^-1^, Brain Heart Infusion, Becton Dickinson, Heidelberg, Germany). For pediocin PA-1 production, the lean GY medium (pH 5.9), which was recently optimized for enhanced peptide yield, was used [86]. Plasmid copy number analyses were conducted in minimal medium that contained per liter: 10 g of glucose, 15 g of (NH_4_)_2_SO_4_, 10 g of casamino acids (Becton Dickinson), 1 g of NaCl, 0.2 g of MgSO_4_ 7H_2_O, 2.55 mg of CaCl_2_, 20 mg of FeSO_4_ 7H_2_O, 0.5 mg of biotin,1 mg of thiamin HCl, 1 mg of calcium panthothenate, 10 mL of a 100 x trace element solution (200 mg L^-1^ FeCl_3_ 6H_2_O, 200 mg L⁻^1^ MnSO_4_ H_2_O, 50 mg L^-1^ ZnSO_4_ 7H_2_O, 20 mg L^-1^ CuCl_2_ 2H_2_O, 20 mg L^-1^ Na_2_B_4_O_7_ 10H_2_O, 10 mg L^-1^ (NH_4_)_6_MO_7_O_24_ 4H_2_O, pH 1.0), 30 µg of 3,4-dihydroxybenzoic acid and 200 mM potassium phosphate (pH 7.8). During the cultivation of inducible production strains, IPTG was added to the culture from a filter-sterilized stock to a final concentration of 0.2 mM. For all media, supplementation with either 50 µg mL^-1^ kanamycin or 12.5 µg mL^-1^ chloramphenicol was used for plasmid maintenance.

###  Genetic and molecular engineering

Design strategies for genome editing and genetic engineering were planned using the SnapGene 7.1.1 software (GSL Biotech, Chicago, IL, USA). Amplification, assembly, purification and transformation of DNA fragments and plasmids were conducted as described elsewhere [87–89]. The promoters *P*_*tuf*_ (200 bp) [90] and *P*_*tac*_ [19] were used for constitutive and IPTG-inducible overexpression of heterologous genes, respectively. Multiple variants of the episomal expression vector pClik *5α* were generated with the aim of increasing plasmid copy number. Mutations were introduced by site-directed mutagenesis, using the QuickChange II XL Site-Directed Mutagenesis Kit (Agilent technologies, Santa Clara, CA, USA) with primers containing the desired change [89]. This approach was used to substitute glycine by l-glutamate at position 429 within the sequence of the RepA protein (RepA^G429E^) and the implementation of a mutation into the promoter region (-10-region) of the *repA*-gene associated antisense-RNA gene *cgrI.* Furthermore, deletion of the putative pClik *5α* partitioning genes (*parAB*) was achieved by dividing the plasmid into fragments lacking the sequence using PCR. Primer-overhangs were introduced for subsequent reassembly of the fragments, forming plasmid pClik *5α* Δ*parAB*. The promoter region *P*_*tac*_ and the *lacI*-repressor gene were deleted from the episomal plasmid pXMJ19 through digestion of the vector using a combination of the restriction enzymes *Sma*I and *Eco*321 (Thermo Fisher Scientific, Waltham, Massachusetts, United States). The plasmid fragments resulting from the enzymatic digestion were then separated via gel electrophoresis. The fragment, lacking *P*_*tac*_ and *lacI* was extracted from the agarose gel and subsequently reassembled to form pXMJ19 Δ*P*_*tac*_ (Δ*P*_*tac*_ Δ*lacI*).

###  Plasmid copy number screening in microbioreactors

Experiments were carried out in 48-well flower plates (MTP-48-B, Beckman Coulter GmbH, Baesweiler, Germany) utilizing a high-throughput microbioreactor for concurrent on-line monitoring of growth and mCherry signals (BioLector I, 1300 rpm, 30 °C, 85% humidity, Beckman Coulter, Baesweiler, Germany). For the initial screen, *C. glutamicum* CR099 strains carrying pClik 5α *P*_*tuf*_ *mCherry*, pCes *P*_*tuf*_ *mCherry*, pXMJ19 Δ*P*_*tac*_ *P*_*tuf*_ *mCherry*, and the different pClik 5α replication variants were cultivated under identical conditions. Each well was filled with 1 mL of medium. The inoculum was cultivated in BHI medium (30 °C, 230 rpm, 10% filling volume). Subsequently, cells were harvested (3 min, 8800 × *g*, room temperature) and transferred to the main culture, conducted in minimal medium, additionally amended with 10 g L^-1^ of casamino acids. Cultures were inoculated to an initial OD_660_ of 0.5. Cell growth and mCherry fluorescence were measured on-line as optical density at 620 nm (OD_620_) and fluorescence (excitation/emission at 580/610 nm) [91].

Estimates on relative plasmid copy numbers were inferred from *mCherry* fluorescence using the chromosomally integrated single-copy strain CR099::Ptuf-mCherry as reference. This fluorescence-ratio approach provides apparent relative gene-dosage estimates rather than absolute plasmid copy numbers, consistent with previous studies [34, 46, 47]. It should be noted that this method may be influenced by factors such as reporter maturation kinetics, promoter activity, and metabolic burden, particularly at high expression levels, and therefore was used primarily for comparative ranking of plasmid variants. As the purpose of the fluorescence-based screening was comparative ranking rather than statistical inference, duplicate biological cultures (*n* = 2) were sufficient to reproducibly resolve relative differences between plasmid variants. To obtain independent molecular validation, plasmid copy number was additionally determined by qPCR as described below.

### qPCR-based plasmid copy number determination

For independent molecular quantification of plasmid copy number, strains harboring the parental pClik 5α *P*_*tuf*_ *mCherry* plasmid and the pClik 5α *repA*^*mu*t^ *Ptuf mCherry* variant were cultivated and sampled at 16 h and 24 h. Total DNA was isolated and subjected to quantitative PCR using *gyrB* as chromosomal single-copy reference gene. Two plasmid-encoded loci, *mCherry* and *kanR*, were quantified in parallel. Relative plasmid copy number was calculated using the 2^−ΔCt^ method, with ΔCt defined as the difference between the Ct value of the plasmid locus and that of *gyrB*. Data represent biological triplicates. Statistical significance was assessed using an unpaired two-sided t-test.

###  Evaluation of producers in microbioreactors

The evaluation of pediocin PA-1 producers was conducted in 48-well flower plates (MTP-48-BOH1, Beckman Coulter GmbH), using a high-throughput microbioreactor with simultaneous on-line monitoring of growth, pH-value and dissolved oxygen (DO) (BioLector I, 700 rpm, 30 °C, 85% humidity, Beckman Coulter) [92]. In each well, 1 mL of GY-medium, buffered at pH 5.9, was employed. The inoculation involved a single preculture grown overnight in BHI medium, utilizing unbaffled shake flasks (10% filling volume) at 30 °C and 230 rpm (Infors HT Multitron, Bottmingen, Switzerland). For inoculation of the main culture, the preculture was harvested by centrifugation (3 min, 8800 *×g*, room temperature), immediately prior to start of the experiment [91]. To minimize the loss of activity due to the oxidation of pediocin PA-1, cultivation supernatants were sampled immediately before the oxygen concentration in the media increased. All experiments were executed in triplicate.

### Quantification of cell density

During experiments in mini-bioreactors, cell concentration was monitored online at 620 nm. The cell concentration of shake-flask based pre-cultures was determined offline by measuring the optical density at 660 nm using a spectrophotometer.

###  Growth inhibition assay for determination of pediocin PA-1 activity

The activity of Pediocin PA-1 in culture supernatants was assessed using growth inhibition assays as previously described [86]. In short, overnight cultures of the sensor strains *L. innocua pIMK2* and *L. innocua pNZ44* were grown in BHI medium in glass tubes filled to 20% capacity and incubated on a rotary shaker at 37 °C and 230 rpm (Infors HT Multitron Samples were serially diluted 2-fold in BHI medium using 96-well plates. The sensor strain was then diluted 1:25 in fresh BHI medium and combined in a 1:1 ratio with the dilution series. The cell density of the sensor strains was measured after incubation on a rotary shaker for 6 h (37 °C, 230 rpm, Infors HT Multitron) using a plate reader at 595 nm (Labsystems iEMS Reader MF, Thermo Fisher, Waltham, MA, USA). Pediocin PA-1 titers are expressed in bacteriocin units per milliliter (BU mL⁻¹), defined as the reciprocal of the highest dilution that achieves 50% inhibition of the sensor strain’s growth [93]. A software-based tool using a sigmoidal dose-response model was employed for parameter estimation, enabling the accurate quantification of pediocin PA-1 levels (Origin 2021, Northampton, UK).

###  Global transcriptome analysis using RNA microarrays

The global expression profile of *C. glutamicum* was analyzed using one-color RNA microarrays as described previously [86, 94]. Customized microarrays (8 × 15 K) were designed using the Agilent Technologies online tool eArray. Each array consisted of 8990 probes covering all annotated genes of *C. glutamicum* ATCC 13,032, in addition to 60 quality control probes (SurePrint G3 Custom GE 8 × 60 K, part number G4863A, Agilent Technologies). Each gene was represented by three distinct probes ranging from 45 to 60 bases in length. Culture samples designated for transcriptome analysis were harvested rapidly (1 min, 13000 *x g*, RT) and immediately transferred into liquid nitrogen. RNA was isolated from the cell pellets using RNeasy Mini Kit (Qiagen, Hilden, Germany), followed by concentration and purity assessment by absorption measurement (NanoDrop 1000, PEQLAB Biotechnology GmbH, Erlangen, Germany). RNA integrity was additionally verified by chip-based electrophoresis (RNA 6000 Nano Kit, 2100 Bioanalyzer System, Agilent Technologies). All samples used for subsequent analysis displayed an RNA integrity number (RIN) of > 9.8.

For labeling, 50 ng of isolated RNA was used as template for the synthesis of Cy3-labeled antisense cRNA (Low Input Quick Amp WT Labeling Kit, Agilent Technologies). Each reaction yielded > 825 ng cRNA with a Cy3 activity of > 15 pmol per µg RNA (NanoDrop 1000, PEQLAB Biotechnology GmbH). Subsequently, 600 ng of labeled cRNA was fragmented and hybridized onto the microarray using the Gene Expression Hybridization Kit (Agilent Technologies). A total volume of 40 µL of the fragmented cRNA solution was loaded between gasket and array slide using the SureHyb chamber (G2534A, Agilent Technologies). Hybridization was carried out in a hybridization oven (G2545A, Agilent Technologies) at 65 °C for 17 h. Arrays were then scanned using a SureScan Microarray Scanner (G4900DA, Agilent Technologies) according to the AgilentG3_GX_1 color scanner protocol.

Raw data were extracted with the Feature Extraction Software (version 12.1.1.1, Agilent Technologies). Data interpretation and visualization were performed using GeneSpring software (version 14.9, Agilent Technologies). Raw microarray data were processed and analyzed by two-way ANOVA in GeneSpring. Differentially expressed genes were identified using a significance threshold of *p* < 0.05 together with an absolute fold-change cutoff of ≥ 2. Transcriptome analysis focused on predefined pairwise comparisons between the native pClik 5α plasmid and the pClik 5α *repA*^*mut*^ derivative, each analyzed in the absence and presence of *mCherry* expression. The two principal comparisons used for biological interpretation in the main text were *repA*^*mut*^ empty vs. native empty and *repA*^*mut*^ *mCherry* vs. native *mCherry*. Additional comparisons addressing the transcriptional impact of *mCherry* expression within each plasmid background were evaluated separately and are provided in the supplementary dataset. To assess the relationship between the two principal comparisons, overlap analyses of significantly up- and downregulated genes were performed after direction-specific filtering based on gene identifiers. Transcriptome results were interpreted primarily at the level of recurring genes and affected functional categories. The complete reprocessed transcriptome dataset is provided in Additional File 2. The microarray dataset has been deposited in the NCBI Gene Expression Omnibus (GEO) under accession number GSE314030.

## Supplementary Information

Below is the link to the electronic supplementary material.

Supplementary Material 1. Complete processed transcriptome dataset.

Supplementary Material 2. Table S1: Sequence of the plasmid pClik 5a MCS in GenBank format. Table S2. Primers used in this work. Figure S1. Plasmid variants harboring different mutations within the origin of replication (ORI) region of pClik 5α. Shown are pClik 5α ΔparAB, in which two ORFs located near the repA gene and identified as potential partitioning genes parA and parB were deleted; pClik 5α repAmut, generated by introducing a point mutation at nucleotide position 1286 (G → A) to substitute glycine with L-glutamate at position 429 of the RepA protein (RepAG429E); and pClik 5α cgrImut, in which two mutations were introduced into the −10 promoter region of the antisense RNA cgrI, specifically (−10) T → C and (−13) A → G. Figure S2. Cultivation profiles of C. glutamicum CR099 strains expressing different plasmid variants carrying the mCherry gene under control of the constitutive Ptuf promoter. Cultivations were performed in a mini-bioreactor. Online optical density measurements at 620 nm were used to monitor cell density, applying a gain factor of 50 (A). mCherry production was monitored online by fluorescence measurements at 580/610 nm (B). Data represent biological duplicates (n = 2). Figure S3. Sequence analysis of the episomal expression vector pClik 5α repAmut Ptuf mCherry isolated from strains with a visually detected loss of mCherry production. Plasmid DNA was isolated from three independent colonies picked from BHI agar plates and subjected to sequencing of the ORI/repA region and the mCherry cassette. Fig. S4: Visual assessment of plasmid stability under non-selective and stress conditions. Cell pellets of C. glutamicum CR099 strains expressing the parental pClik 5α Ptuf mCherry plasmid (parent), the high-copy variant pClik 5α repAmut Ptuf mCherry (repAmut), and the double-mutant pClik 5α repAmut cgrImut Ptuf mCherry (repAmut cgrImut) are shown under different cultivation conditions. (A) Cultivation in GY medium without kanamycin (non-selective conditions). (B) Cultivation in minimal medium supplemented with kanamycin, imposing additional metabolic stress. While the parental and repAmut plasmids maintained stable mCherry-associated fluorescence, the double-mutant construct exhibited reduced and variable fluorescence across conditions, indicating impaired plasmid stability that is exacerbated under non-selective and stress conditions. Figure S5. Estimation of plasmid copy number based on mCherry fluorescence signals. For each strain, mCherry fluorescence was plotted against biomass during exponential growth to derive a linear relationship. Slopes were obtained by linear regression and normalized to the slope of the single gene-copy strain CR099::Ptuf mCherry, which served as reference, to estimate relative plasmid copy numbers. Figure S6. Cultivation profile and sampling scheme for global transcriptome analysis of C. glutamicum CR099 strains expressing the high-copy plasmid pClik 5α repAmut. The experimental setup included cultivation and sampling of CR099 strains harboring either the empty pClik 5α repAmut plasmid or pClik 5α repAmut-based expression of the mCherry gene under control of the constitutive Ptuf promoter. RNA samples of strains harboring the native pClik 5α plasmid, either empty or carrying Ptuf-driven mCherry, were used as references for the transcriptome analysis. Time points for RNA sampling are marked. Experiments were conducted in mini-bioreactors under standardized cultivation conditions (1300 rpm, 30°C). Data represent biological triplicates (n = 3). Figure S7: Transcriptome analysis of mCherry-dependent transcriptional responses. (A) Number of significantly up- and downregulated genes identified by two-way ANOVA (p < 0.05, absolute fold change ≥ 2) in comparisons of mCherry-expressing versus empty plasmid backgrounds for both repAmut and native plasmids. Data represent biological triplicates (n = 3). (B) Overlap of significantly regulated genes between the two comparisons shown as Venn diagrams for upregulated (left) and downregulated (right) genes. Only genes with consistent regulation direction were considered. (C) Distribution of differentially expressed genes across major functional categories based on the same statistical thresholds. Compared to the repA-driven response (Fig. 3), mCherry-associated transcriptional changes are more context-dependent and exhibit limited overlap between conditions. Figure S8. Principal component analysis (PCA) of gene expression datasets obtained from microarray analyses. Shown are: (A) C. glutamicum CR099 expressing the empty plasmid pClik 5α repA with C. glutamicum CR099 carrying pClik 5α as reference; (B) C. glutamicum CR099 expressing the mCherry gene from pClik 5α compared to the empty pClik 5α variant; and (C) C. glutamicum CR099 expressing the mCherry gene from pClik 5α repAmut with C. glutamicum CR099 carrying pClik 5α as reference.

## Data Availability

The datasets supporting the conclusions of this article are included within the article and its supplementary materials (Additional File 1 and Additional File 2). The complete processed transcriptome dataset has been deposited in the NCBI Gene Expression Omnibus (GEO) under accession number GSE314030.
