# Supplementary material for: High-copy plasmid engineering enhances recombinant protein and antimicrobial peptide production in Corynebacterium glutamicum
Source: Microb Cell Fact. 2026 Apr 13;25:101. doi: 10.1186/s12934-026-03004-y (PMC13088423; doi:10.1186/s12934-026-03004-y)
Supplement: Supplementary file 2 — Supplementary Material 2. Table S1: Sequence of the plasmid pClik 5a MCS in GenBank format. Table S2. Primers used in this work. Figure S1. Plasmid variants harboring different mutations within the origin of replication (ORI) region of pClik 5α. Shown are pClik 5α ΔparAB, in which two ORFs located near the repA gene and identified as potential partitioning genes parA and parB were deleted; pClik 5α repAmut, generated by introducing a point mutation at nucleotide position 1286 (G → A) to substitute glycine with L-glutamate at position 429 of the RepA protein (RepAG429E); and pClik 5α cgrImut, in which two mutations were introduced into the −10 promoter region of the antisense RNA cgrI, specifically (−10) T → C and (−13) A → G. Figure S2. Cultivation profiles of C. glutamicum CR099 strains expressing different plasmid variants carrying the mCherry gene under control of the constitutive Ptuf promoter. Cultivations were performed in a mini-bioreactor. Online optical density measurements at 620 nm were used to monitor cell density, applying a gain factor of 50 (A). mCherry production was monitored online by fluorescence measurements at 580/610 nm (B). Data represent biological duplicates (n = 2). Figure S3. Sequence analysis of the episomal expression vector pClik 5α repAmut Ptuf mCherry isolated from strains with a visually detected loss of mCherry production. Plasmid DNA was isolated from three independent colonies picked from BHI agar plates and subjected to sequencing of the ORI/repA region and the mCherry cassette. Fig. S4: Visual assessment of plasmid stability under non-selective and stress conditions. Cell pellets of C. glutamicum CR099 strains expressing the parental pClik 5α Ptuf mCherry plasmid (parent), the high-copy variant pClik 5α repAmut Ptuf mCherry (repAmut), and the double-mutant pClik 5α repAmut cgrImut Ptuf mCherry (repAmut cgrImut) are shown under different cultivation conditions. (A) Cultivation in GY medium without kanamycin (non-selective conditi [file 12934_2026_3004_MOESM2_ESM.docx]

**Additional file 1 to**

**High-copy plasmid engineering enhances recombinant protein and antimicrobial peptide production in *Corynebacterium glutamicum***

Jens Christmann^1^, Peng Cao^1^, Michael Kohlstedt^1^, Oliver Goldbeck^2^, Christian U. Riedel^2^, Judith Becker^1^, and Christoph Wittmann^1*^

^1^ Institute for Systems Biotechnology, Saarland University, Saarbrücken, Germany

^2^ Institute of Microbiology and Biotechnology, University of Ulm, Ulm, Germany

**Table S1: Sequence of the plasmid pClik 5a MCS in GenBank format.**

LOCUS Exported 5106 bp ds-DNA circular SYN 30-MAR-2026

DEFINITION synthetic circular DNA.

ACCESSION .

VERSION .

KEYWORDS pClik_5aMCS

SOURCE synthetic DNA construct

ORGANISM synthetic DNA construct

REFERENCE 1 (bases 1 to 5106)

AUTHORS .

TITLE Direct Submission

JOURNAL Exported Mar 30, 2026 from SnapGene Viewer 4.1.9

http://www.snapgene.com

COMMENT GenBank 5091 bp CIRCULAR 19-FEB-2014

FEATURES Location/Qualifiers

source 1..5106

/organism="synthetic DNA construct"

/mol_type="other DNA"

misc_feature 12..83

/label=MCS

misc_feature 470..1261

/gene="KanR"

/label=kanR

/note="/ugene_group=""CDS"""

/note="/ugene_name=""KanR"""

misc_feature 1528..2388

/gene="ORI"

/label=ORI E.coli (pMB1)

misc_feature 2534..3211

/label=ORF

misc_feature 3172..3244

/label=cgrI

misc_feature 3249..4708

/label=repA

ORIGIN

1 tcgatttaaa tctcgagagg cctgacgtcg ggcccggtac cacgcgtcat atgactagtt

61 cggacctagg gatatcgtcg acatcgatgc tcttctgcgt taattaacaa ttgggatcct

121 ctagacccgg gatttaaatg atccgctagc gggctgctaa aggaagcgga acacgtagaa

181 agccagtccg cagaacggtg ctgacccgga tgaatgtcag ctactgggct atctggacag

241 ggaaaacgca agcgcaaaga gaaagcaggt agcttgcagt gggcttacat ggcgatagct

301 agactgggcg gttttatgga cagcaagcga accggaattg ccagctgggg cgccctctgg

361 taaggttggg aagccctgca aagtaaactg gatggctttc ttgccgccaa ggatctgatg

421 gcgcagggga tcaagatctg atcaagagac aggatgagga tcgtttcgca tgattgaaca

481 agatggattg cacgcaggtt ctccggccgc ttgggtggag aggctattcg gctatgactg

541 ggcacaacag acaatcggct gctctgatgc cgccgtgttc cggctgtcag cgcaggggcg

601 cccggttctt tttgtcaaga ccgacctgtc cggtgccctg aatgaactgc aggacgaggc

661 agcgcggcta tcgtggctgg ccacgacggg cgttccttgc gcagctgtgc tcgacgttgt

721 cactgaagcg ggaagggact ggctgctatt gggcgaagtg ccggggcagg atctcctgtc

781 atctcacctt gctcctgccg agaaagtatc catcatggct gatgcaatgc ggcggctgca

841 tacgcttgat ccggctacct gcccattcga ccaccaagcg aaacatcgca tcgagcgagc

901 acgtactcgg atggaagccg gtcttgtcga tcaggatgat ctggacgaag agcatcaggg

961 gctcgcgcca gccgaactgt tcgccaggct caaggcgcgc atgcccgacg gcgaggatct

1021 cgtcgtgacc catggcgatg cctgcttgcc gaatatcatg gtggaaaatg gccgcttttc

1081 tggattcatc gactgtggcc ggctgggtgt ggcggaccgc tatcaggaca tagcgttggc

1141 tacccgtgat attgctgaag agcttggcgg cgaatgggct gaccgcttcc tcgtgcttta

1201 cggtatcgcc gctcccgatt cgcagcgcat cgccttctat cgccttcttg acgagttctt

1261 ctgagcggga ctctggggtt cgaaatgacc gaccaagcga cgcccaacct gccatcacga

1321 gatttcgatt ccaccgccgc cttctatgaa aggttgggct tcggaatcgt tttccgggac

1381 gccggctgga tgatcctcca gcgcggggat ctcatgctgg agttcttcgc ccacgctagc

1441 ggcgcgccgg ccggcccggt gtgaaatacc gcacagatgc gtaaggagaa aataccgcat

1501 caggcgctct tccgcttcct cgctcactga ctcgctgcgc tcggtcgttc ggctgcggcg

1561 agcggtatca gctcactcaa aggcggtaat acggttatcc acagaatcag gggataacgc

1621 aggaaagaac atgtgagcaa aaggccagca aaaggccagg aaccgtaaaa aggccgcgtt

1681 gctggcgttt ttccataggc tccgcccccc tgacgagcat cacaaaaatc gacgctcaag

1741 tcagaggtgg cgaaacccga caggactata aagataccag gcgtttcccc ctggaagctc

1801 cctcgtgcgc tctcctgttc cgaccctgcc gcttaccgga tacctgtccg cctttctccc

1861 ttcgggaagc gtggcgcttt ctcatagctc acgctgtagg tatctcagtt cggtgtaggt

1921 cgttcgctcc aagctgggct gtgtgcacga accccccgtt cagcccgacc gctgcgcctt

1981 atccggtaac tatcgtcttg agtccaaccc ggtaagacac gacttatcgc cactggcagc

2041 agccactggt aacaggatta gcagagcgag gtatgtaggc ggtgctacag agttcttgaa

2101 gtggtggcct aactacggct acactagaag gacagtattt ggtatctgcg ctctgctgaa

2161 gccagttacc ttcggaaaaa gagttggtag ctcttgatcc ggcaaacaaa ccaccgctgg

2221 tagcggtggt ttttttgttt gcaagcagca gattacgcgc agaaaaaaag gatctcaaga

2281 agatcctttg atcttttcta cggggtctga cgctcagtgg aacgaaaact cacgttaagg

2341 gattttggtc atgagattat caaaaaggat cttcacctag atccttttaa aggccggccg

2401 cggccgcgca aagtcccgct tcgtgaaaat tttcgtgccg cgtgattttc cgccaaaaac

2461 tttaacgaac gttcgttata atggtgtcat gaccttcacg acgaagtacc aaaattggcc

2521 cgaatcatca gctatggatc tctctgatgt cgcgctggag tccgacgcgc tcgatgctgc

2581 cgtcgattta aaaacggtga tcggattttt ccgagctctc gatacgacgg acgcgccagc

2641 atcacgagac tgggccagtg ccgcgagcga cctagaaact ctcgtggcgg atcttgagga

2701 gctggctgac gagctgcgtg ctcggcagcg ccaggaggac gcacagtagt ggaggatcga

2761 atcagttgcg cctactgcgg tggcctgatt cctccccggc ctgacccgcg aggacggcgc

2821 gcaaaatatt gctcagatgc gtgtcgtgcc gcagccagcc gcgagcgcgc caacaaacgc

2881 cacgccgagg agctggaggc ggctaggtcg caaatggcgc tggaagtgcg tcccccgagc

2941 gaaattttgg ccatggtcgt cacagagctg gaagcggcag cgagaattat ccgcgatcgt

3001 ggcgcggtgc ccgcaggcat gacaaacatc gtaaatgccg cgtttcgtgt ggccgtggcc

3061 gcccaggacg tgtcagcgcc gccaccactt gcaccgaatc ggcagcagcg tcgcgcgtcg

3121 aaaaagcgca caggcggcaa gaagcgataa gctgcacgaa tacctgaaaa atgttgaacg

3181 ccccgtgagc ggtaactcac agggcgtcgg ctaaccccca gtccaaacct gggagaaagc

3241 gctcaaaaat gactctagcg gattcacgag acattgacac accggcctgg aaattttccg

3301 ctgatctgtt cgacacccat cccgagctcg cgctgcgatc acgtggctgg acgagcgaag

3361 accgccgcga attcctcgct cacctgggca gagaaaattt ccagggcagc aagacccgcg

3421 acttcgccag cgcttggatc aaagacccgg acacgggaga aacacagccg aagttatacc

3481 gagttggttc aaaatcgctt gcccggtgcc agtatgttgc tctgacgcac gcgcagcacg

3541 cagccgtgct tgtcctggac attgatgtgc cgagccacca ggccggcggg aaaatcgagc

3601 acgtaaaccc cgaggtctac gcgattttgg agcgctgggc acgcctggaa aaagcgccag

3661 cttggatcgg cgtgaatcca ctgaagcggg aaatgccagc tcatctggct cattgatccg

3721 gtgtatgccg cagcaggcat gagcagcccg aatatgcgcc tgctggctgc aacgaccgag

3781 gaaatgaccc gcgttttcgg cgctgaccag gctttttcac ataggctgag ccggtggcca

3841 ctgcacgtct ccgacgatcc caccgcgtac cgctggcatg cccagcacaa tcgcgtggat

3901 cgcctagctg atcttatgga ggttgctcgc atgatctcag gcacagaaaa acctaaaaaa

3961 cgctatgagc aggagttttc tagcggacgg gcacgtatcg aagcggcaag aaaagccact

4021 gcggaagcaa aagcacttgc cacgcttgaa gcaagcctgc cgagcgccgc tgaagcgtct

4081 ggagagctga tcgacggcgt ccgtgtcctc tggactgctc cagggcgtgc cgcccgtgat

4141 gagacggctt ttcgccacgc tttgactgtg ggataccagt taaaagcggc tggtgagcgc

4201 ctaaaagaca ccaagatcat cgacgcctac gagcgtgcct acaccgtcgc tcaggcggtc

4261 ggagcagacg gccgtgagcc tgatctgccg ccgatgctgt gaccgccaga cgatggcgcg

4321 acgtgtgcgc ggctacgtcg ctaaaggcca gccagtcgtc cctgctcgtc agacagagac

4381 gcagagcagc cgagggcgaa aagctctggc cactatggga agacgtggcg gtaaaaaggc

4441 cgcagaacgc tggaaagacc caaacagtga gtacgcccga gcacagcgag aaaaactagc

4501 taagtccagt caacgacaag ctaggaaagc taaaggaaat cgcttgacca ttgcaggttg

4561 gtttatgact gttgagggag agactggctc gtggccgaca atcaatgaag ctatgtctga

4621 atttagcgtg tcacgtcaga ccgtgaatag agcacttaag tctgcgggca ttgaacttcc

4681 acgaggacgc cgtaaagctt cccagtaaat gtgccatctc gtaggcagaa aacggttccc

4741 cccgtagggg tctctctctt ggcctccttt ctaggtcggg ctgattgctc ttgaagctct

4801 ctaggggggc tcacaccata ggcagataac ggttccccac cggctcacct cgtaagcgca

4861 caaggactgc tcccaaagat cttcaaagcc actgccgcga ctccgcttcg cgaagccttg

4921 ccccgcggaa atttcctcca ccgagttcgt gcacacccct atgccaagct tctttcaccc

4981 taaattcgag agattggatt cttaccgtgg aaattcttcg caaaaatcgt cccctgatcg

5041 cccttgcgac gttgctcgcg gtcggtgccg ctggttgcgc ttggcttgac cgacttgagc

5101 ggccgc

**//**

**Table S2:** Primers used in this work

| **Primer** | **5’-Sequence-3’** | **Plasmid** |
| --- | --- | --- |
| **Primers used for the construction of episomal and integrative transformation plasmids** | | |
| PR_1 _sense_ | GCTCTTCTGCGTTAATTAACAATTGGGATCCTCTAGACCCTGGCCGTTACCCTGCGAA | *pClik 5α^repA^ P_tuf_ pedACD^Cg^*  *pClik 5α^cgrl^ P_tuf_ pedACD^Cg^*  *pClik 5α^repA cgrl^ P_tuf_ pedACD^Cg^* |
| PR_2 _anti-sense_ | CGCTTCCTTTAGCAGCCCGCTAGCGGATCATTTAAATCCCTTATTCCTGGTTATGAATGAGGCGTGC |  |
| PR_3 _sense_ | GAAGTCCAGGAGGACATACAATGAAGAAGATTGAGAAGCTGACCGA |  |
| PR_4 _anti-sense_ | AGCTTCTCAATCTTCTTCATTGTATGTCCTCCTGGACTTCGT |  |
| PR_1 _sense_ | AATTGGGATCCTCTAGACCCTGGCCGTTACCCTGCGAATG | *pClik 5α P_tuf_ mCherry*  *pClik 5α^repA^ P_tuf_ mCherry*  *pClik 5α^cgrl^ P_tuf_ mCherry*  *pClik 5α^repA cgrl^ P_tuf_ mCherry* |
| PR_2 _anti-sense_ | TCCTCGCCCTTGCTCACCATTGTATGTCCTCCTGGACTTC |  |
| PR_1 _sense_ | GAAGTCCAGGAGGACATACAATGGTGAGCAAGGGCGAGGA |  |
| PR_2 _anti-sense_ | TAGCGGATCATTTAAATCCCTTACTTGTACAGCTCGTCCA |  |
| PR_1 _sense_ | AGATGGGTACCGAGCTCGAATGGCCGTTACCCTGCGAATG | *pXMJ19*^Δ^*^Ptac^ P_tuf_ mCherry* |
| PR_2 _anti-sense_ | GCCAAAACAGCCAAGCTGAATTATTCCTGGTTATGAATGA |  |
| PR_1 _sense_ | AATTGGGATCCTCTAGACCCTCACCGCCTGGCCCTGAGAG | *pClik 5α^repA^ P_tac_ mCherry* |
| PR_2 _anti-sense_ | ACTCTAGAGAAGGAGTTTTC |  |
| PR_3 _sense_ | GAAAACTCCTTCTCTAGAGT |  |
| PR_4 _anti-sense_ | CTAGCGGATCATTTAAATCCCTTATTCCTGGTTATGAATG |  |
| PR_1 _sense_ | AATTGGGATCCTCTAGACCCTAAGCAATGGCCTACAACCA | *pClik int sacB P_tuf_ mCherry* |
| PR_2 _anti-sense_ | CATTCGCAGGGTAACGGCCAGGTTTATTTCCCTTTAACTG |  |
| PR_1 _sense_ | AATTGGGATCCTCTAGACCCTAAGCAATGGCCTACAACCA |  |
| PR_2 _anti-sense_ | CGAAGGCACGGTGTTCACGATTACTTGTACAGCTCGTCCA |  |
| PR_1 _Fra3_ _sense_ | TGGACGAGCTGTACAAGTAATCGTGAACACCGTGCCTTCG |  |
| PR_2 _Fra3 anti-sense_ | TAGCGGATCATTTAAATCCCACGCATGAGTGTGCTTGTGG |  |
| **Primers used for plasmid integration side sequencing** | | |
| PR_1 _sense_ | GTGGCCGACAATCAATGAAGCTATG | *pClik 5α* |
| PR_2 _anti-sense_ | CCGGAGAACCTGCGTGCAATCCAT |  |
| PR_1 _sense_ | AATAATAGTGAACGGCAGGT | *pClik int sacB* |
| PR_2 _anti-sense_ | ATTGTCTGTTGTGCCCAGTCATAG |  |
| PR_1 _sense_ | GAGCATTGAGAAAGCGCCAC | *pXMJ19* |
| PR_2 _anti-sense_ | GCGGCGGTGTTCGCGGGTAA |  |
| **Primers used in XL Site-Directed Mutagenesis Kit (Agilent Technologies)** | | |
| PR_1 _sense_ | ACAAGCTAGGAAAGCTAAAGAAAATCGCTTGACCATTGCA | *pClik 5α ^repA^* |
| PR_2 _anti-sense_ | TGCAATGGTCAAGCGATTTTCTTTAGCTTTCCTAGCTTGT |  |
| PR_1 _sense_ | AGAAAGCGCTCAAAAATGACCCTGGCGGATTCACGAGACA | *pClik 5α ^cgrl^* |
| PR_2 _cgrl bottom_ | TGTCTCGTGAATCCGCCAGGGTCATTTTTGAGCGCTTTC |  |
| **Primers used for the deletion of potential partitioning genes *parA* and *parB*** | | |
| PR_1 _sense_ | GCCGCGCAAAGTCCCGCTTCCGCGCGTCGAAAAAGCGCAC | *pClik 5 α*^Δ^*^parAB^ P_tuf_ mCherry pClik 5 α*^Δ^*^parAB repA^ P_tuf_ mCherry*  *pClik 5 α*^Δ^*^parAB cgrl^ P_tuf_ mCherry*  *pClik 5 α*^Δ^*^parAB repA cgrl^ P_tuf_ mCherry* |
| PR_2 _anti-sense_ | GTGCGCTTTTTCGACGCGCGGAAGCGGGACTTTGCGCGGC |  |
| PR_3 _sense_ | GGCTTGACCGACTTGAGCGG |  |
| PR_4 _anti-sense_ | CCGCTCAAGTCGGTCAAGCC |  |
| PR_5 _sense_ | GCCTTATCCGGTAACTATCG |  |
| PR_6 _anti-sense_ | CGATAGTTACCGGATAAGGC |  |


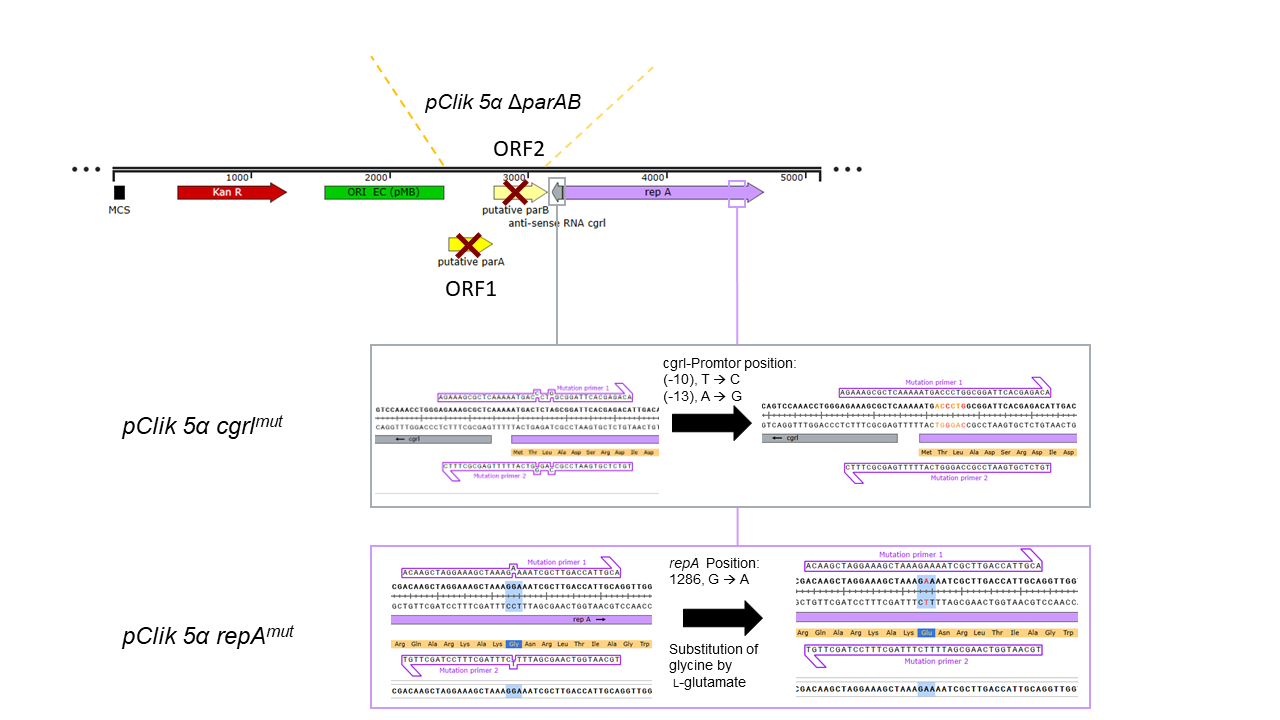


**Figure S1.** Plasmid variants harboring different mutations within the origin of replication (ORI) region of pClik 5α. Shown are pClik 5α *ΔparAB*, in which two ORFs located near the *repA* gene and identified as potential partitioning genes *parA* and *parB* were deleted; pClik 5α *repA^mut^*, generated by introducing a point mutation at nucleotide position 1286 (G → A) to substitute glycine with L-glutamate at position 429 of the RepA protein (RepAG429E); and pClik 5α *cgrI^mut^*, in which two mutations were introduced into the −10 promoter region of the antisense RNA cgrI, specifically (−10) T → C and (−13) A → G.





**Figure S2.** Cultivation profiles of *C. glutamicum* CR099 strains expressing different plasmid variants carrying the *mCherry* gene under control of the constitutive *P_tuf_* promoter. Cultivations were performed in a mini-bioreactor. Online optical density measurements at 620 nm were used to monitor cell density, applying a gain factor of 50 (A). mCherry production was monitored online by fluorescence measurements at 580/610 nm (B). Data represent biological duplicates (n = 2).


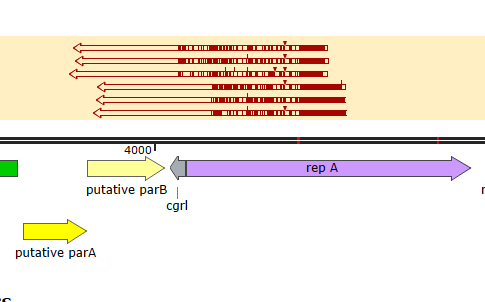


**Figure S3.** Sequence analysis of the episomal expression vector pClik 5α *repA^mut^ P_tuf_ mCherry* isolated from strains with a visually detected loss of mCherry production. Plasmid DNA was isolated from three independent colonies picked from BHI agar plates and subjected to sequencing of the ORI/repA region and the mCherry cassette.


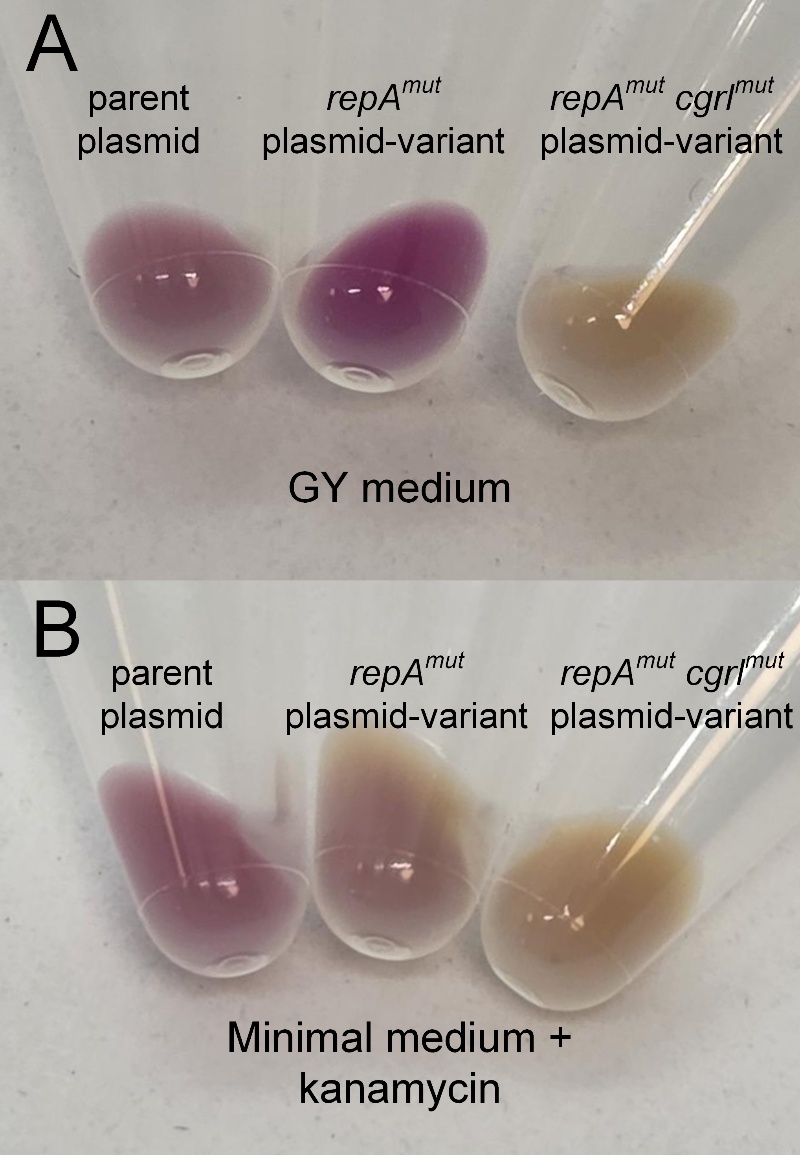


**Fig. S4: Visual assessment of plasmid stability under non-selective and stress conditions.** Cell pellets of *C. glutamicum* CR099 strains expressing the parental pClik 5α *P_tuf_ mCherry* plasmid (parent), the high-copy variant pClik 5α *repA^mut^ P_tuf_ mCherry* (*repA^mut^*), and the double-mutant pClik 5α *repA^mut^ cgrI^mut^ P_tuf_ mCherry* (*repA^mut^ cgrI^mut^*) are shown under different cultivation conditions. (A) Cultivation in GY medium without kanamycin (non-selective conditions). (B) Cultivation in minimal medium supplemented with kanamycin, imposing additional metabolic stress. While the parental and *repA^mut^* plasmids maintained stable mCherry-associated fluorescence, the double-mutant construct exhibited reduced and variable fluorescence across conditions, indicating impaired plasmid stability that is exacerbated under non-selective and stress conditions.





**Figure S5.** Estimation of apparent plasmid copy number based on mCherry fluorescence signals. For each strain, mCherry fluorescence was plotted against biomass during exponential growth to derive a linear relationship. For the estimation, slopes were obtained by linear regression and normalized to the slope of the single gene-copy strain CR099::*P_tuf_ mCherry*, which served as reference.





**Figure S6.** The experimental setup included cultivation and sampling of CR099 strains harboring either the empty pClik 5α *repA^mut^* plasmid or pClik 5α *repA^mut^*^-^based expression of the *mCherry* gene under control of the constitutive *P_tuf_* promoter. RNA samples of strains harboring the native pClik 5α plasmid, either empty or carrying *P_tuf_*-driven mCherry, were used as references for the transcriptome analysis. Time points for RNA sampling are marked. Experiments were conducted in mini-bioreactors under standardized cultivation conditions (1300 rpm, 30°C). Data represent biological triplicates (n = 3).

**Figure S7: Transcriptome analysis of *mCherry*-dependent transcriptional responses.** (A) Number of significantly up- and downregulated genes identified by two-way ANOVA (p < 0.05, absolute fold change ≥ 2) in comparisons of *mCherry*-expressing versus empty plasmid backgrounds for both repAmut and native plasmids. Data represent biological triplicates (n = 3). (B) Overlap of significantly regulated genes between the two comparisons shown as Venn diagrams for upregulated (left) and downregulated (right) genes. Only genes with consistent regulation direction were considered. (C) Distribution of differentially expressed genes across major functional categories based on the same statistical thresholds. Compared to the *repA*-driven response (Fig. 3), *mCherry*-associated transcriptional changes are more context-dependent and exhibit limited overlap between conditions.


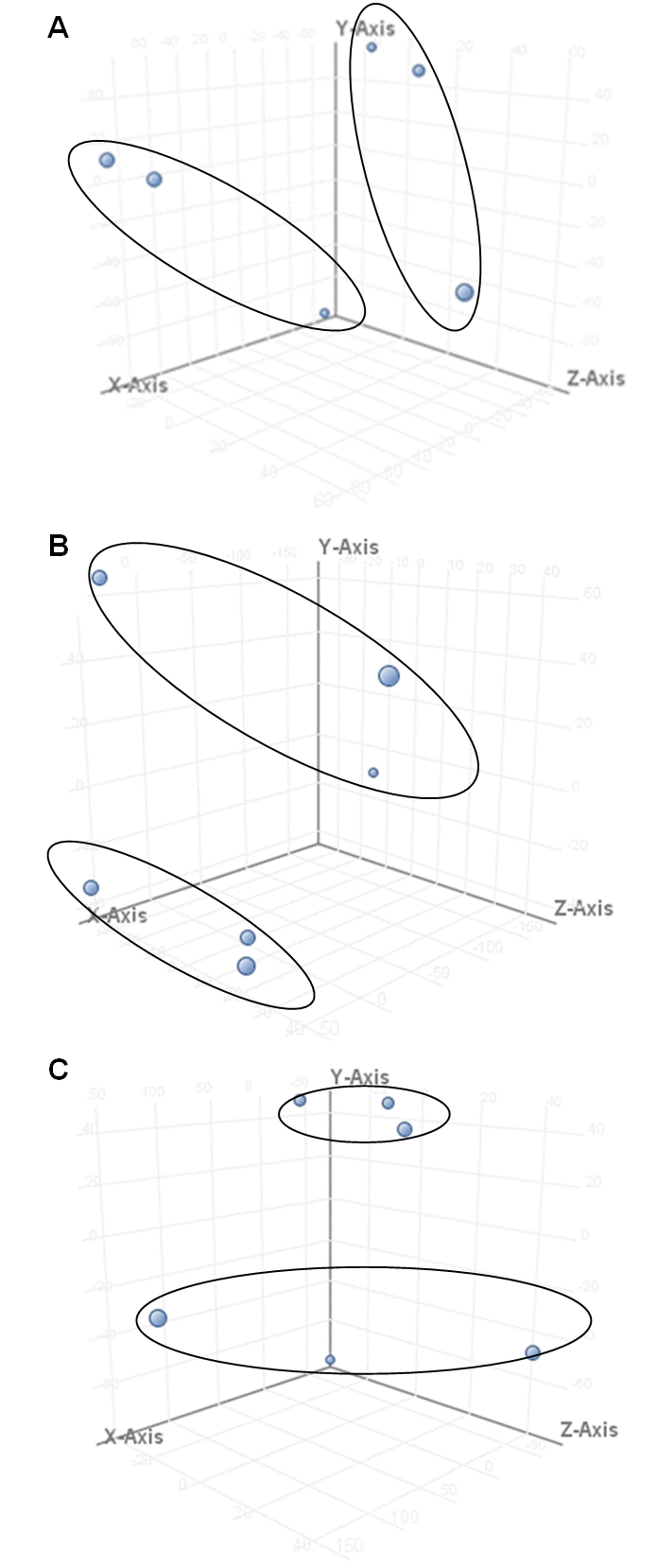


**Figure S8.** Principal component analysis (PCA) of gene expression datasets obtained from microarray analyses. Shown are: (A) *C. glutamicum* CR099 expressing the empty plasmid pClik 5α *repA* with *C. glutamicum* CR099 carrying pClik 5α as reference; (B) *C. glutamicum* CR099 expressing the *mCherry* gene from pClik 5α compared to the empty pClik 5α variant; and (C) *C. glutamicum* CR099 expressing the *mCherry* gene from pClik 5α *repA^mut^* with *C. glutamicum* CR099 carrying pClik 5α as reference.
